# Migration, Foraging, and Residency Patterns for Northern Gulf Loggerheads: Implications of Local Threats and International Movements

**DOI:** 10.1371/journal.pone.0103453

**Published:** 2014-07-30

**Authors:** Kristen M. Hart, Margaret M. Lamont, Autumn R. Sartain, Ikuko Fujisaki

**Affiliations:** 1 Southeast Ecological Science Center, U.S. Geological Survey, Davie, Florida, United States of America; 2 Southeast Ecological Science Center, U.S. Geological Survey, Gainesville, Florida, United States of America; 3 Scientific R&D, Support to U.S. Geological Survey Southeast Ecological Science Center, Cherokee Nation Technology, Solutions, LLC, Davie, Florida, United States of America; 4 Ft. Lauderdale Research and Education Center, University of Florida, Davie, Florida, United States of America; UC Santa Cruz Department of Ecology and Evolutionary Biology, United States of America

## Abstract

Northern Gulf of Mexico (NGoM) loggerheads (*Caretta caretta*) make up one of the smallest subpopulations of this threatened species and have declining nest numbers. We used satellite telemetry and a switching state-space model to identify distinct foraging areas used by 59 NGoM loggerheads tagged during 2010–2013. We tagged turtles after nesting at three sites, 1 in Alabama (Gulf Shores; n = 37) and 2 in Florida (St. Joseph Peninsula; n = 20 and Eglin Air Force Base; n = 2). Peak migration time was 22 July to 9 August during which >40% of turtles were in migration mode; the mean post-nesting migration period was 23.0 d (±13.8 d SD). After displacement from nesting beaches, 44 turtles traveled to foraging sites where they remained resident throughout tracking durations. Selected foraging locations were variable distances from tagging sites, and in 5 geographic regions; no turtles selected foraging sites outside the Gulf of Mexico (GoM). Foraging sites delineated using 50% kernel density estimation were located a mean distance of 47.6 km from land and in water with mean depth of −32.5 m; other foraging sites, delineated using minimum convex polygons, were located a mean distance of 43.0 km from land and in water with a mean depth of −24.9 m. Foraging sites overlapped with known trawling activities, oil and gas extraction activities, and the footprint of surface oiling during the 2010 Deepwater Horizon oil spill (n = 10). Our results highlight the year-round use of habitats in the GoM by loggerheads that nest in the NGoM. Our findings indicate that protection of females in this subpopulation requires both international collaborations and management of threats that spatially overlap with distinct foraging habitats.

## Introduction

Many highly mobile marine vertebrates, including turtles, make long distance migrations to breeding areas. The locations of marine turtle breeding and nesting sites are often up to hundreds to thousands of kilometers from foraging areas in which animals are resident for the majority of their lives [1], [2], [3]. Foraging resources contribute towards fat stores that allow females to attain sufficient body condition to sustain reproductive migrations and an energetically demanding reproductive season. While at foraging grounds, female marine turtles recover from the previous nesting season and build energy reserves for vitellogenesis [4]. All clutches of follicles that will support the subsequent nesting season are developed at this time using resources available at the foraging site [4]. Thus, characteristics of foraging grounds can impact various aspects of reproduction [5]–[7]. Therefore, identifying the locations of these key foraging areas and characterizing the habitat and resources contained therein is a critical step in ensuring population persistence and recovery [8].

Satellite tracking has emerged as a strong tool for uncovering oceanic routes taken during marine animal migrations, as well as areas of residence at-sea. Recently, analyses of multiple long-term tracking datasets have contributed to a broad understanding of marine animal habitat use across taxa at regional scales [9,], and for particular taxa (i.e., turtles [10], [11]) at global scales. By combining many years of tracking data for post-nesting Kemp’s ridleys (*Lepidochelys kempii*) in the Gulf of Mexico (GoM), Shaver et al. [12] discovered important at-sea locations where high numbers of adult female Kemp’s ridleys are resident at foraging areas. For adult female loggerhead marine turtles (*Caretta caretta*) in particular, combined data sets from multiple subpopulations in the Northwest Atlantic have produced a more comprehensive understanding of movement patterns and foraging sites used by breeding individuals [2], [13]–[16]. For loggerheads in the GoM, Hart et al. [2] delineated 2 common coastal foraging areas (i.e., southwest Florida and off Mexico) used by adult females from 3 separate subpopulations. Foley et al. [14], [15] later confirmed the importance of these 2 areas with additional tracks from turtles in 2 of the same 3 subpopulations. Results from these two studies, though conducted over a decade apart, show how multi-year tracking datasets can illuminate foraging areas that are repeatedly used and therefore of high conservation significance. Understanding the relationships among nesting groups and foraging areas is necessary for population-level assessments of anthropogenic threats and design of conservation strategies [17], [18].

### Loggerheads in the Northwest Atlantic

Loggerhead marine turtles in the Northwest Atlantic are listed as threatened under the U.S. Endangered Species Act. The species exists as five subpopulations [17] and 10 management units [19], [20] based on mitochondrial DNA analyses. The subpopulations in the Dry Tortugas (DRTO) and Northern Gulf of Mexico (NGoM) are the two smallest, with individual nesting subpopulation estimates of 258–496 females (50 percentile distribution = 331) and 323–634 females (50 percentile distribution = 432), respectively [21]. Currently, declining nest abundance in the NGoM subpopulation [22], [23] may indicate an overall decline in the number of individuals; trends for the DRTO subpopulation are unknown.

Declines in nest abundance for the NGoM subpopulation [23] may be attributed to several factors, one of which is consistent interactions with commercial fisheries, a threat that pressures all Northwest Atlantic loggerheads [24]–[26]. In addition to direct mortality in trawling gear, several studies have shown that shrimp trawling can damage benthic habitat and reduce invertebrate abundance [27]–[29]. Because loggerheads forage frequently on benthic invertebrates [30], these activities may reduce loggerhead prey. The most recent Biological Opinion from National Marine Fisheries Service [31] forecasts approximately 4,000 loggerhead deaths annually in U.S. waters due to the shrimp trawling fishery. Most shrimp trawling occurs along the continental shelf in waters less than −18 m deep during April through October, a period that coincides with nesting activity and migratory movements for loggerhead turtles [2], [32], [33]. In addition to these long-standing threats to loggerheads in the GoM, a new and serious threat emerged in the NGoM in 2010 when the Deepwater Horizon (DWH) oil platform exploded resulting in the largest oil spill in U.S. history [34], [35]. This disaster resulted in oil pollution that may damage many species of marine plants and animals, as well as their habitats [35]. Such a regional incident could drive this relatively small NGoM subpopulation of loggerheads [21] towards extinction.

Previous loggerhead tracking studies where turtles were tagged on beaches in the NGoM [2], [14], [15] identified foraging grounds only in the GoM; no loggerhead from the NGoM subpopulation, that we are aware of, has ever been tracked to a foraging ground outside the GoM. However, sample sizes of turtles tagged on beaches in the NGoM in those studies have been relatively small (n≤14). Thus, our objectives in this study on adult female loggerheads nesting in the NGoM were to: (1) characterize migration time, (2) delineate and characterize foraging sites, (3) examine foraging sites in relation to turtle size, (4) assess overlap of foraging sites with anthropogenic threats (i.e., shrimp trawling, oil and gas platforms, and surface oiling of DWH oil spill), and (5) synthesize our results for this NGoM subpopulation and compare to previously published data.

## Materials and Methods

### Ethics statement

This work was conducted under the authority of research permits from the Florida Fish and Wildlife Conservation Commission Marine Turtle Permit #094, Bon Secour National Wildlife Refuge Special Use Permit #12-006S (issued to K. Hart), Federal U.S. Fish and Wildlife Permit #TE206903-1 (issued to J. Phillips), as well as the U.S Geological Survey Animal Care and Use permit #SESC-2011-05.

### Study sites

**Figure 1:**
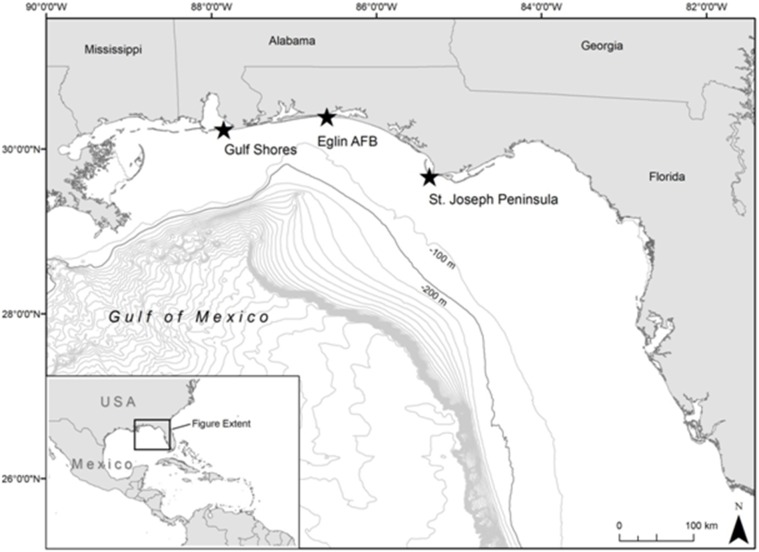
Study sites (stars) in the Northern Gulf of Mexico where adult female loggerheads (*Caretta caretta*) were satellite-tagged from 2010–2013 in Alabama (Gulf Shores) and Florida (Eglin Air Force Base and St. Joseph Peninsula). Bathymetry contour lines are shown in 100 m intervals with 200 m shown as a darker line to indicate approximate end of neritic zone and bounds of depth filter used in analysis.

Turtle tagging occurred at three study sites in the NGoM (Figure 1). The Gulf Shores, Alabama (GS) site included the Perdue Unit of the U.S. Fish and Wildlife Service Bon Secour National Wildlife Refuge and adjacent private lands in Baldwin County. The Florida sites comprised approximately 17 km of beach along the St. Joseph Peninsula (SJP) and 18 km of beach owned by Eglin Air Force Base (EAFB) on Santa Rosa Island in Northwest Florida (NWFL). These locations represent the approximate eastern (SJP), middle (EAFB) and western (GS) extents of known loggerhead nesting in the NGoM [17] and are separated by approximately 250 km (straight line distance).

### Turtle capture and transmitter deployment

In GS, nightly surveys were conducted from 9 pm to 6 am every day from 1 June to ∼30 June. On SJP, nightly surveys were conducted from 9 pm to 6 am every day from 15 May to 15 Aug. On EAFB, nightly surveys were conducted from 9 pm to 6 am for one week in July 2012 (10 to 17 July). We used 59 satellite transmitters to monitor the movements of 59 post-nesting loggerhead turtles in the GoM over a 4 year period between 2010 and 2013; details on those tagged in 2010 were previously published in Hart et al. [2], but we include them in our analyses here. In addition, one loggerhead was tagged in both 2011 and 2012 in GS, and is considered as two separate turtles for tracking analysis, and two loggerheads were tagged using the same tag in 2012 because of interception of one turtle on a southwest Florida nesting beach; see Table S1).

Turtles were documented and outfitted with transmitters using established protocols [36]. Turtle interception and tagging followed methods identical to those in Hart et al. [33]. Briefly, we intercepted loggerhead females after they had finished nesting on the beach. Immediately after marking each turtle with Inconel and PIT tags, we took standard carapace measurements, including curved (CCL) and straight (SCL) carapace lengths. We adhered platform transmitter terminals (PTTs) using slow-curing epoxy and used several types of PTTs (Table S2). We streamlined attachment materials to minimize drag effects [37] on the turtle’s swimming ability and limited the epoxy footprint. Each tag was set to be active for 24 h d^1^ from June–November, then every 3^rd^ day from November–May. We compared mean turtle size (SCL-tip) in two tagging locations, Alabama and Florida, with t-test.

### Marine turtle tracking and filtering

Location data were filtered using Satellite Tracking and Analysis Tool (STAT; [38]) available on www.seaturtle.org. Location classes (LC) 3, 2, 1, 0, A, and B were used to reconstruct routes and calculate straight-line and total distances that the turtles traveled. Locations were rejected if they were LC Z (for which no error estimation was available). Argos assigns accuracy estimates of <250 m for LC 3, 250 to <500 m for LC 2, 500 to <1500 m for LC 1, and >1500 m for LC 0 [39]. The estimated accuracy is unknown for LCs A and B (but these LCs can be useful; see [40], [41], and locations failing the Argos plausibility tests are tagged as class LC Z. Both traditional least-squares location processing (2010) as well as Kalman-filtering (initiated in 2011; [42]) of location data was performed by Argos. This newly-implemented Kalman-filtering algorithm provides more estimated positions and significantly improves position accuracy, most significantly for locations obtained in LCs A and B [43].

### Switching state-space modeling

Location data obtained through satellite transmitters are often received at irregular time intervals, and sometimes involve large gaps and positional errors. Ad hoc filtering of location data based on location quality is not sufficient to remove erroneous locations and also results in loss of information [44]. Switching state-space modeling (SSM; [45], [46]) has two components, accounting for location errors (observation error) and the animal’s behavior [47], [48]. The observational error is based on the location quality class from Argos data. The two-state switching correlated random walk models the transition between the two behavioral states (see [47] for more detailed description of model), with the observation equation translating observed satellite locations to true unobserved locations at equal time intervals. The model [47] has previously been applied to model movement of marine animals including marine mammals [49] (blue whales; *Balaenoptera musculus*), and several species of turtles [2], [12], [33], [39], [46], [50]–[54].

We used a switching SSM approach to determine the beginning and end date of migration and foraging periods for each turtle following Hart et al. [2]. Earlier applications defined a binary behavioral model with ‘foraging’ and ‘migration’ [47], [50], [51]; however, since we tagged animals during the nesting season, our definitions for behavioral modes were ‘foraging and/or inter-nesting’ and ‘migration’. However, we only summarized data for the periods after migration away from nesting beaches, and then during time periods with ‘foraging’ locations. The model predicted a value of “2” for migration and “1” for non-migration. It is possible for some predicted locations to be unassigned (“1.4”) to either migration or foraging and/or inter-nesting; we did not use any unassigned points in home range estimates We applied a model used by Breed et al. [48], which is a modified version of a model described by Jonsen et al. [47] (for equations see [55]) that estimates model parameters by Markov Chain Monte Carlo (MCMC) using WinBUGS via the software program R. We fit the model to tracks of each individual turtle to estimate location and behavioral state every eight hours from two independent and parallel chains of MCMC samples. Our samples from the posterior distribution were based on 10,000 samples after a burn-in of 7000 samples and thinning the remaining samples by five. The convergence was monitored by observing model parameters of two independent chains that were mixed in the trace plots as suggested by Breed et al. [48]. We summarized data until the transmitters stopped sending information or until the time of data synthesis (15 October 2013). With the beginning and end dates for foraging and migration determined by the SSM, we used original, filtered satellite locations from within those time periods for further analysis.

### Migration periods

After fitting the switching SSM to individual loggerhead tracks, we identified locations where turtles were in migration mode. For this analysis, we summarized migration periods after inter-nesting periods and before arrival at foraging grounds. To do this, we used the migration period directly before the foraging period (see: In-water foraging areas section below for details on determining arrival at foraging areas). We then visually confirmed these migration periods with location data and summarized migration both temporally (number of days) and spatially.

### Foraging areas

From the foraging periods determined with SSM, we filtered out locations requiring speeds >5 kph, along with any other obviously erroneous locations (on land, spatially very distant, etc.). We also removed locations deeper than −200 m (neritic zone cutoff); Hawkes et al. [13] found that adult female loggerheads in the southeast U.S. did not generally leave the waters of the continental shelf (within −200 m). Additionally, for all tracks in this study with potential foraging periods (i.e., those with SSM output and foraging locations, n = 44; see Results), the removed depth locations accounted for less than 1% of available locations after speed filtering.

If an individual foraging period was at least 20 days in length, we generated mean daily locations using the filtered locations to minimize autocorrelation; the resulting coordinates provided raw data for kernel density estimation (KDE). Kernel density is a non-parametric method used to identify one or more areas of disproportionately heavy use (i.e., core areas) within a home-range boundary [56]–[58], with appropriate weighting of outlying observations. We used the Home-Range Tools for ArcGIS extension [59] and fixed-kernel least-squares cross-validation smoothing factor (h*_cv_*) for each KDE [60], [61]. When we observed unequal variance of the x and y coordinates, we rescaled the data to select the best bandwidth, following Seaman and Powell [61] and Laver and Kelly [62]. We used ArcGIS 9.3 [63] to calculate the in-water area (km^2^) within each kernel density contour (50% and 95%) and to plot the data; we used 95% KDEs to represent the overall home foraging range, and the 50% KDEs to represent core area of activity at foraging sites [64].

For foraging periods that did not qualify for KDE analysis(those without 20 mean daily locations) but at least six days long, we performed minimum convex polygon (MCP) analysis (100% of points; [65], [66]) using ArcMap 9.3 [63] and obtained the in-water area for these. See Methods S1 for exceptions.

We also tested location data for and quantified site-fidelity to in-water foraging locations using the Animal Movement Analysis Extension for ArcView 3.2. Using Monte Carlo Random Walk simulations (100 replicates), we tested tracks during the foraging period for spatial randomness against randomly generated walks [64]. We bounded the range for random walks from −200 m to 0 m bathymetry to include only the realistic extent of the in-water habitat for our animals during the study period; however, we smoothed out the North Gulf shoreline with a 2000 m buffer to account for many small bays and points close to land. Tracks exhibiting site-fidelity indicate movements that are more spatially constrained rather than randomly dispersed [64].

### Foraging area characteristics

We calculated the centroid of each MCP and 50% KDE; if a 50% KDE included multiple activity centers, we calculated the centroid of the largest activity center. We extracted depths for all centroids and the distance from each to the nearest land. For bathymetry, we used the NOAA National Geophysical Data Center (GEODAS) ETOPO1, 1 arc-minute global relief model of Earth’s surface (http://www.ngdc.noaa.gov/m,gg/geodas/geodas.html; accessed 26 January 2012). We also plotted 2 KDE foraging centroids for loggerheads tagged in the NGoM and previously published in Hart et al. [2] for comparison.

For turtles with short tracking durations (i.e., those that were not long enough to determine foraging location), and those that failed to run successfully in SSM (i.e., often due to large gaps in transmissions), we plotted the last transmitted, filtered location. We also plotted last filtered locations for turtles that had KDEs or MCPs but for which site-fidelity tests failed. We plotted these “last points” for a visual representation of general areas these turtles occupied either during migration or foraging. We conducted simple regression analysis to examine association between turtle size and each of size of 50% KDE and bathymetry of foraging site. We also used simple regression analysis to examine whether size of 50% KDE is associated with number of tracking days.

### Geographic regions

We assigned foraging sites into 5 geographic regions, following H. Vander Zanden (pers. comm.), and similar to VanderZanden et al. [7], Arendt et al. [67], and Pajuelo et al. [68]: (1) Western GoM (WGoM) that extends from the Texas/Mexico border to the tip of the peninsula in Plaquemines Parish, Louisiana where the Mississippi River drains into the GoM border [69], [70]; (2) NGoM that extends from the tip of the peninsula in Plaquemines Parish, Louisiana east to Apalachee Bay in Florida [71]; (3) Western Florida (WFL) that extends from Apalachee Bay, Florida to the northern boundary of the Florida Keys; (4) Subtropical Northwest Atlantic (SNWA) that extends from the Florida Keys, around the tip of Florida and north to West Palm Beach, Florida (following Pajuelo et al. [72]); and (5) Southern GoM (SGoM) that encompasses the area off the Yucatan Peninsula, Mexico and is what is geologically known as the Campeche Bank. Using an ANOVA, we tested whether mean turtle size (SCL-tip) varied by region. We compared proportions of turtles from each nesting beach by regions, using a Chi-squared test. We performed statistical tests in R, with α  = 0.05 to assess significance.

### Foraging ‘hotspots’

To depict all foraging locations used by turtles over time, we calculated the number of turtle-foraging days in grid cells (10×10 km); the grid extended across the extent of the GoM within the −200 m isobath. For each turtle track with days in foraging mode, we counted number of days each turtle was observed (turtle days) in each grid cell during foraging periods using all satellite locations except for LC Z.

### Foraging areas and anthropogenic activities

Finally, to help provide guidance for conservation and management actions with foraging habitat, we also mapped the overlap of commercial shrimp trawling during 2011–2012 (sum of days fished over the two years), the locations of active oil and gas platforms, and the oiling extent from the DWH oil spill. We created a layer in ArcGIS 9.3 using shrimp trawling data and statistical zone cutoffs provided by NOAA (J. Nance & A. Frick, pers. comm.). We determined the number of shrimp trawling days in each area as it related to the centroids. The layer for oil and gas platforms was obtained from http://www.data.boem.gov/homepg/data_center/mapping/geographic_mapping.asp, accessed on 4 December 2013. Platforms in this layer included all platforms existing in the U.S. Bureau of Ocean and Energy Management (BOEM) database. To relate centroids to the DWH oil spill, we counted the number of foraging centroids located within the surface oiling layer from (Environmental Response Management Application, Web application, ERMA Deepwater Gulf Response, National Oceanic and Atmospheric Administration, 2014. Web.19 December 2013. See http://response.restoration.noaa.gov/erma/. Data URL: http://gomex.erma.noaa.gov/erma.html#/x=-88.25810&y=27.03211&z=6&layers=23037 (Downloaded 9 January, 2014). In addition, we determined how many centroids were within state, U.S. federal or international waters. We obtained layers on 16 December 2013 for the Exclusive Economic Zone (EEZ; version 7, updated 20 November 2012, from http://www.marineregions.org/downloads.php) and State Submerged Lands (SSL; http://www.marinecadastre.gov/Data/default.aspx and http://www.fgdl.org/metadataexplorer/explorer.jsp).

## Results

### Turtles

From 2011–2013, we recorded 55 tracks of 54 individuals (one turtle tracked twice as 106345 in 2011 and 119944 in 2012, hereafter considered two separate turtles for all analyses). In 2010, we tracked 4 turtles and those 4 tracks were analyzed and published in Hart et al. [2]; however, to provide the most comprehensive view of the spatial distribution of loggerheads from the NGoM, we combined the 4 tracks from 2010 with the 55 tracks from 2011–2013 for a total of 59 tracks (Table 1; 2010 turtles treated in all summaries like other turtles, see Methods S1 and supporting information in Methods S1 for more information). Turtles (n = 59) ranged in size from 82.4–104.3 cm curved carapace length (CCL-tip; mean ± SD = 95.0±4.9 cm, Table S2). Mean turtle sizes were similar across study sites (t_30.3_ = 1.066, p = 0.295). In a total of 9229 tracking days, individual tracking durations for all 59 turtles ranged from 24 to 582 d (mean ± SD = 156.4±126.9 d; Table S2).

**Table 1:** Summary of satellite-tracking details for adult foraging loggerheads (*Caretta caretta*) in the Northern Gulf of Mexico (n = 59).

	Year				Size (CCL-tip, cm)	
	2010	2011	2012	2013	mean	SD
Gulf Shores, Alabama	0	13	10	14	94.4	3.8
St. Joseph Peninsula, Florida	4	0	10	6		
Eglin Air Force Base, Florida	0	0	2	0	96.0*	6.3*
TOTAL	4	13	22	20	95.0	4.9

*mean and SD for all Florida turtles from both sites.

### Spatial analysis summary

We attempted to run each of 59 turtle tracks through SSM; however, we dropped 13 tracks due to short tracking durations or large transmission gaps. Four of the turtles were originally analyzed in Hart et al. [2]; we added tracking days for 2 of these 4 turtles for this analysis; see Methods S1. In sum, we obtained SSM results for 46 turtles and used these for further analysis, such as KDEs and/or MCPs (see below, Foraging areas). For 15 turtles (13 with no SSM results and 2 with SSM results but that failed site fidelity tests), we plotted their last points.

### Migration periods

**Figure 2:**
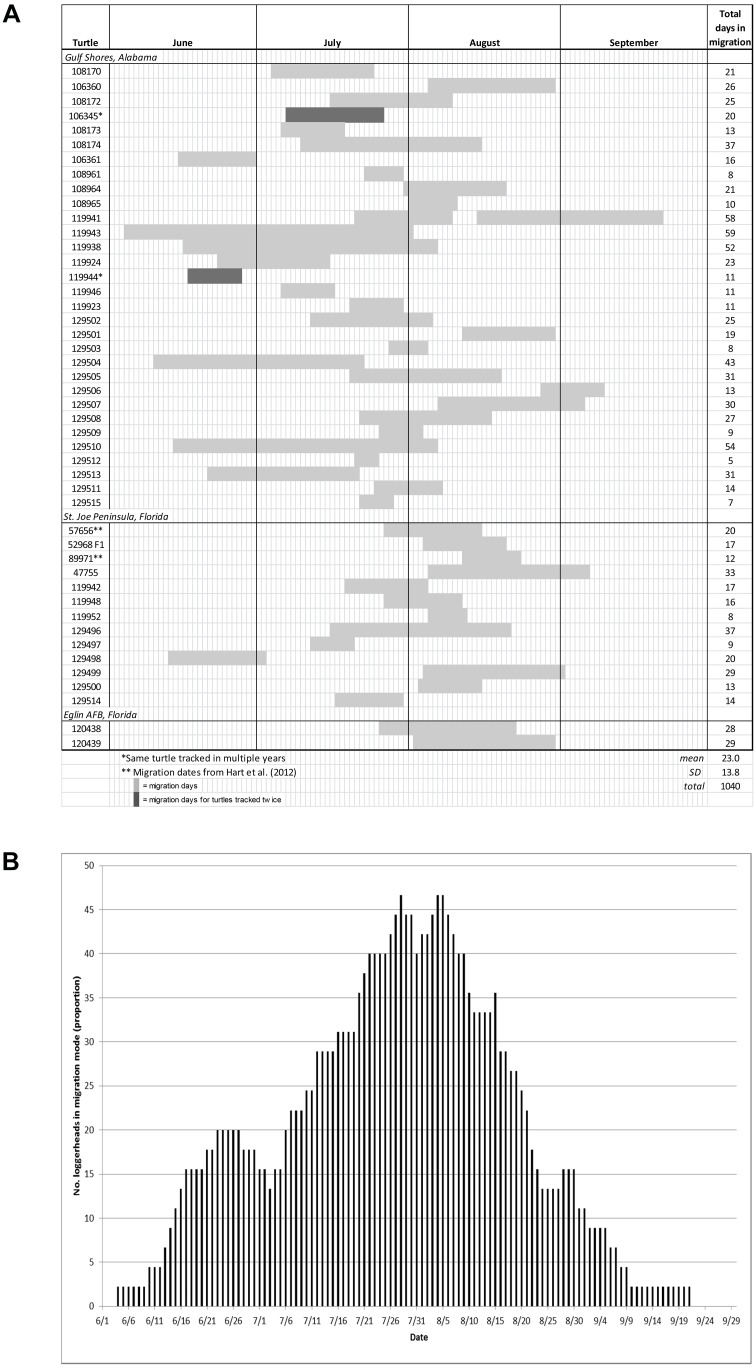
Migration between inter-nesting sites and foraging areas for adult female loggerheads (*Caretta caretta*) tagged in the Northern Gulf of Mexico. Panel A: Total migration days and periods. Panel B: proportion of turtles in migration mode during tracking periods.

We considered the migration period directly before the foraging period to be the main migration period; this classification was appropriate for all but 3 turtles (see supporting information in Methods S1, and Figure S1; this classification also included the previously tracked turtles from 2010). Forty-six turtles were tracked from their inter-nesting site through migration to their foraging grounds; migration periods across all turtles totaled 1040 days (Figure 2 and S2). Migration duration ranged from 5–59 days (mean ± SD = 23.0±13.8 d), with a peak in migration timing of 7/22–8/9 during which >40% of turtles were in migration mode (Figure 2).

### Foraging areas

As stated above, SSM was successful for 46 turtles (see Figures S1 and S3 and Table S3 for example SSM prediction paths and associated model parameters for turtles 119946, 119941, 129506 and 129515). This total number of tracks included reanalyzed tracks from 2 of the 2010 turtles that transmitted beyond the cutoff date used in Hart et al. [2] (see Supporting Information (Methods S1) and 2 previously published tracks (Figure S4). Of the 44 new tracks, 36 turtles had foraging periods with a successful KDE analysis, resulting in 38 KDEs (turtles 120439 and 52968 each had 2 foraging sites, a ‘primary’ and a ‘secondary’ as in Foley et al. [15]; Figure S5). All KDE foraging areas combined (this study and the 2 previously published in Hart et al. [2], n = 40 KDEs) totaled 5339 days tracked in foraging and ranged from 22–416 d (mean ± SD = 133.5±117.1 d; Table 2). All of these displayed site fidelity to their foraging areas and represented 18,938 filtered locations (Table 2). Of these filtered locations, we obtained 3181 total mean daily locations for analyses. The overall size of 50% core-use areas during foraging ranged from 4.5–851.8 km^2^ (mean ± SD = 100.7±141.8 km^2^; Table 2). Overall home range size (95% KDE) ranged from 22.0–3628.5 km^2^ (mean ± SD = 504.3±621.4 km^2^). Turtle size and size of 50% KDE were not significantly associated (F_1,36_ = 0.511, p-value = 0.4792, R^2^ = 0.014) and the size of 50% KDEs were not influenced by tracking duration (F_1,36_ = 0.258, p-value = 0.6146, R^2^ = 0.007).

**Table 2:** Kernel density estimation (KDE) results for adult female loggerhead (*Caretta caretta*) turtles during the foraging period.

Tag Number	First Foraging Date	Last Foraging Date (days)	Filtered Locations	Mean Daily Locations	50% KDE area (sq km)	95% KDE area (sq km)	Foraging Region
***Gulf Shores, AL***							
108170	7/25/2011	3/1/2012 (221)	487	78	97.6	534.3	NGoM
106360	8/31/2011	12/24/2011 (116)	196	39	127.2	721.8	WFL
108172	8/10/2011	2/25/2012 (200)	447	109	206.1	875.9	WFL
106345*	7/27/2011	11/10/2011 (107)	204	35	56.6	276.0	NGoM
108173	7/19/2011	4/2/2012 (259)	659	123	31.9	190.7	WFL
108174	8/16/2011	11/7/2011 (84)	141	31	83.2	357.5	WFL
106361	7/1/2011	6/18/2012 (354)	744	116	851.8	3628.5	NGoM
108964	8/21/2011	9/14/2011 (25)	149	25	330.5	1432.0	WFL
108965	8/11/2011	7/21/2012 (346)	1166	170	84.7	685.9	WFL
119941	9/26/2012	8/31/2013 (340)	359	64	10.4	78.4	SNWA
119943	8/2/2012	11/20/2012 (111)	273	37	25.9	168.6	WFL
119938	8/7/2012	2/22/2013 (200)	529	98	31.6	181.0	WFL
119924	7/16/2012	9/10/2012 (57)	426	57	115.0	584.6	NGoM
119944*	6/28/2012	7/15/2013 (383)	775	125	52.3	292.2	NGoM
119923	7/31/2012	1/7/2013 (161)	741	112	25.8	120.8	WFL
129502	8/6/2013	10/15/2013 (71)	941	71	56.9	222.2	SNWA
129504	7/23/2013	10/14/2013 (84)	507	84	249.3	1452.3	WFL
129505	8/20/2013	10/15/2013 (57)	815	57	7.8	48.6	WFL
129506	9/10/2013	10/15/2013 (36)	209	36	170.7	898.4	WFL
129507	9/6/2013	10/15/2013 (40)	707	40	26.9	106.3	SGoM
129508	8/18/2013	10/15/2013 (59)	444	59	4.5	22.0	WFL
129510	8/7/2013	10/14/2013 (69)	419	69	38.2	189.4	NGoM
129511	8/8/2013	9/23/2013 (47)	270	47	106.8	496.3	NGoM
129512	7/26/2013	10/15/2013 (82)	670	82	44.0	194.7	WGoM
129515	8/9/2013	9/22/2013 (45)	298	45	38.1	177.8	NGoM
129513	7/22/2013**	8/12/2013 (22)	201	22	55.2	238.1	NGoM
***St. Joseph Peninsula, FL***							
57656	8/15/2010	9/12/2010 (29)	52	15***	229.4	911.7	SGoM
52968 F1	8/21/2010	9/29/2011 (405)	801	363	75.8	1037.6	WFL
52968 F2	10/3/2011	3/6/2012 (156)	266	134	34.6	305.4	WFL
89971	8/23/2010	9/16/2010 (25)	133	25	53.8	236.8	WFL
47755	9/7/2010	1/4/2011 (120)	325	114	47.9	307.1	SGoM
119942 F2	9/4/2012	9/29/2012 (26)	121	26	186.4	893.5	NGoM
129497	7/21/2013	10/12/2013 (84)	540	79	5.2	26.8	WFL
129498	7/3/2013	10/15/2013 (105)	547	60	34.2	134.8	NGoM
129499	9/2/2013	10/15/2013 (44)	296	44	105.8	489.4	SGoM
129500	8/16/2013	10/15/2013 (61)	327	61	77.4	418.7	WFL
129514	7/31/2013	10/15/2013 (77)	701	77	26.6	105.3	WFL
***Eglin AFB, FL***							
120439	8/31/2012	1/9/2013 (132)	443	65	77.8	366.3	SGoM
120439 F2	1/14/2013	4/6/2013 (83)	126	32	77.1	309.4	SGoM
120438	8/24/2012	10/13/2013 (416)	1483	255	67.2	453.9	SGoM
*mean*		*133.5*	*473.5*	*79.5*	*100.7*	*504.3*	
*SD*		*117.1*	*306.8*	*65.5*	*141.8*	*621.4*	

Foraging dates based on switching state-space model (SSM) results; KDE areas include in-water area only. F1 or F2 indicates first (F1) or second (F2) foraging areas for a particular turtle. Turtles with no SSM or foraging periods not included. Filtered locations are from within foraging period only. Foraging region abbreviations are as in text. All foraging locations listed here passed site fidelity with p>99.0099, except for 129506 (passed with p>98.0198)

*same turtle tracked/observed in 2011 and 2012.

**foraging date is earlier than 15 August cut-off for this turtle because she migrated to foraging ground early.

***mean daily location less than 20 because part of different study.

We calculated 10 MCPs for 7 turtles (Table 3; Figure S6), including one (turtle 108171) with the foraging period not determined by SSM, as described in the supporting information (Methods S1). All 10 turtles with MCPs displayed site fidelity and the time periods extended from 6 to 62 d (not every tracking day resulted in a location; mean ± SD = 26.0±20.4 d; Table 3) and the size of MCPs ranged from 148.5–1987.1 km^2^ (mean ± SD = 648.4±562.3 km^2^; Table 3).

**Table 3:** Minimum Convex Polygon (MCP) areas for adult female loggerhead (*Caretta caretta*) foraging periods; MCP areas include in-water area only.

**Tag Number**	**First Foraging Date**	**Last Foraging Date**	**Filtered Locations**	**Site Fidelity** *	**MCP area (sq km)**	**Foraging Region**
***Gulf Shores, AL***						
108171	9/4/2012	9/20/2012 (17)	33	p>96.0396	205.2	SGoM
108961	7/31/2011	8/17/2011 (18)	233	p>99.0099	199.1	NGoM
119946	7/17/2012	9/4/2012 (50)	243	p>99.0099	796.9	NGoM
***St. Joseph Peninsula, FL***						
119948	8/12/2012	10/12/2012 (62)	194	p>99.0099	148.5	WFL
119952	8/13/2012	10/3/2012 (52)	124	p>98.0198	1987.1	NGoM
129496 (F1)	8/22/2013	9/5/2013 (15)	47	p>99.0099	809.5	SGoM
129496 (F2)	9/13/2013	9/19/2013 (7)	16	p>98.0198	238.7	SGoM
129496 (F3)	9/28/2013	10/15/2013 (18)	92	p>99.0099	911.8	SGoM
***Eglin AFB***						
120439 (F3)	4/18/2013	4/23/2013 (6)	18	p>98.0198	355.4	SGoM
120439 (F4)	5/10/2013	5/24/2013 (15)	62	p>99.0099	832.2	SGoM
*mean*		*26.0*	*106.2*		*648.4*	
*SD*		*20.4*	*88.0*		*562.3*	

Foraging region abbreviations are as in text.

*All MCPs here passed the site fidelity test.

### Foraging area characteristics

**Figure 3:**
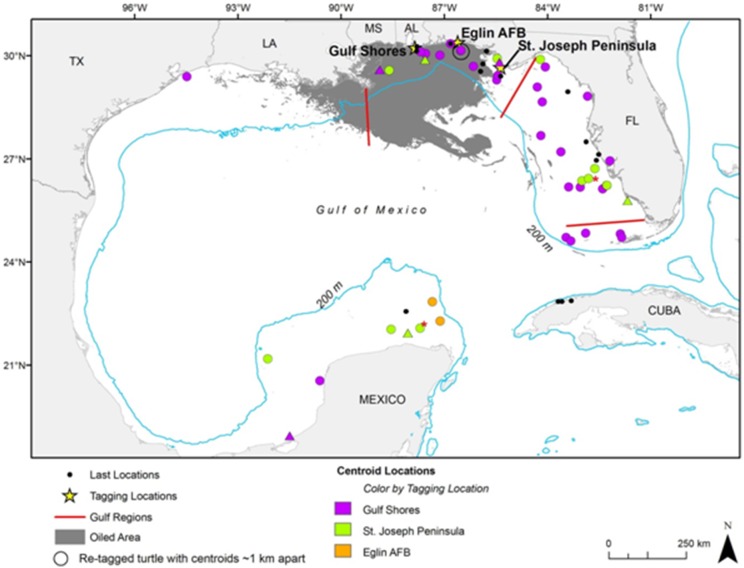
Kernel density estimation (KDE; circles) and minimum convex polygon (MCP; triangles) centroids for 44 adult female loggerheads (*Caretta caretta*) satellite-tagged in the Northern Gulf of Mexico (42 turtles with KDEs and/or MCPs and 2 from previous study Hart et al. (2012)). If a turtle had more than one centroid, only one is shown, with the KDE or MCP with the most points chosen and KDEs given priority. Turtles with centroids removed include: turtle 129496, only MCP F3 shown; turtle 120439, only KDE F1 shown; and turtle 52968, only KDE F1 shown. Two centroids are from Hart et al. (2012) and are shown with a red asterisk to the top right of the centroid. The larger hollow circle depicts where two KDE centroids overlap for the same turtle tagged in both 2011 (tag 106345) and 2012 (tag 119944). Also shown are the last transmitted, filtered locations for 15 turtles (small black dots); all of these last locations are from turtles tagged in Gulf Shores, Alabama. Gulf regional separations are shown as red lines (see Figure S5 for more information). Oil layer: Environmental Response Management Application, Web application, ERMA Deepwater Gulf Response, National Oceanic and Atmospheric Administration, 2014, Web, 19 December 2013. See http://response.restoration.noaa.gov/erma/. Data URL: http://gomex.erma.noaa.gov/erma.html#/x=-88.25810&y=27.03211&z=6&layers=23037 Downloaded 9 January 2014.

We calculated centroids for the 38 KDEs and 10 MCPs (Figure 3) for turtles tracked from 2011–2013 (see Methods S1 for 2010 turtles). Distances to the nearest land from centroids of 50% KDEs ranged from 0.6–138.4 km (mean ± SD = 47.6±38.9 km; Table 4). Bathymetry values (i.e., a proxy for water depths) at these centroid locations ranged from −72.0 to −2.0 m (mean ± SD = −32.5±19.8 m; Table 4) at all sites. Distances to the nearest land from centroids of the 10 MCPs ranged from 1.6–136.5 km (mean ± SD = 43.0±50.9 km; Table 4). Bathymetry values at these locations ranged from −65.0 to −2.0 m (mean ± SD = −24.9±22.1 m; Table 4). Turtle size was not significantly associated with bathymetry of foraging site (F_1,42_ = 1.416, p-value = 0.2407 R^2^ = 0.033).

**Table 4:** Minimum Convex Polygon (MCP) and Kernel density estimation (KDE) centroid characteristics (depth, distance, region, trawling, jurisdiction, oiling) for adult female loggerheads (*Caretta caretta*) during the foraging period.

Gulf Shores, AL									
108170	KDE		−21	4.1	NGoM	9248.8	3	Florida	1
106360	KDE		−64	52.8	SNWA	48.8	1	USA	
108172	KDE		−66	38.6	SNWA	48.8	1	USA	
106345*	KDE		−32	25.8	NGoM	9248.8	3	USA	1
108171	MCP		−10	8.4	SGoM	N/A	N/A	Mexico	
108173	KDE		−46	90.2	WFL	9248.8	3	USA	
108174	KDE		−44	20.9	SNWA	9248.8	3	USA	
106361	KDE		−28	22.7	NGoM	2502.8	2	USA	1
108961	MCP		−7	1.6	NGoM	2095.7	2	Florida	
108964	KDE		−58	120.4	WFL	48.8	1	USA	
108965	KDE		−14	36.4	WFL	1143.3	2	USA	
119941	KDE		−11	8.9	SNWA	1143.3	2	Florida	
119943	KDE		−29	80.6	WFL	9248.8	3	USA	
119938	KDE		−22	42.8	WFL	9248.8	3	USA	
119924	KDE		−36	34.8	NGoM	2502.8	2	USA	1
119944*	KDE		−32	25.0	NGoM	9248.8	3	USA	1
119946	MCP		−8	10.3	NGoM	6815.0	3	USA	1
119923	KDE		−37	111.4	WFL	9248.8	3	USA	
129502	KDE		−18	21.3	SNWA	1143.3	2	USA	
129504	KDE		−52	90.1	WFL	9248.8	3	USA	
129505	KDE		−2	6.3	WFL	1143.3	2	Florida	
129506	KDE		−63	128.9	WFL	48.8	1	USA	
129507	KDE		−3	12.5	SGoM	N/A	N/A	Mexico	
129508	KDE		−2	0.6	WFL	2095.7	2	Florida	
129510	KDE		−44	42.3	NGoM	9248.8	3	USA	
129512	KDE		−16	14.2	NGoM	6815.0	3	USA	1
129513	KDE		−12	12.3	WGoM	10108.0	4	Texas	
129511	KDE		−48	61.3	NGoM	9248.8	3	USA	1
129515	KDE		−27	27.5	NGoM	9248.8	3	USA	
**St. Joseph Peninsula, FL**									
57656**	KDE		−33	58.9	SGoM	N/A	N/A	Mexico	
52968 F1	KDE		−16	26.6	WFL	1143.3	2	USA	
52968 F2	KDE		−15	27.0	WFL	1143.3	2	USA	
89971**	KDE		−31	67.4	WFL	9248.8	3	USA	
47755	KDE		−30	54.2	SGoM	N/A	N/A	Mexico	
119942 F2	KDE		−28	31.3	NGoM	2502.8	2	USA	1
119948	MCP		−2	7.2	WFL	1143.3	2	Florida	
119952	MCP		−37	41.4	NGoM	2502.8	2	USA	1
129496 (F1)	MCP		−20	25.2	SGoM	N/A	N/A	Mexico	
129496 (F2)	MCP		−20	28.6	SGoM	N/A	N/A	Mexico	
129296 (F3)	MCP		−20	34.2	SGoM	N/A	N/A	Mexico	
129497	KDE		−6	12.6	WFL	1143.3	2	Florida	
129498	KDE		−6	3.8	NGoM	1143.3	2	Florida	
129499	KDE		−64	28.9	SGoM	N/A	N/A	Mexico	
129500	KDE		−41	78.5	WFL	9248.8	3	USA	
129514	KDE		−23	33.1	WFL	9248.8	3	USA	
**Eglin AFB, FL**									
120438	KDE		−45	74.0	SGoM	N/A	N/A	Mexico	
120439 (F1)	KDE		−62	138.4	SGoM	N/A	N/A	Mexico	
120439 (F2)	KDE		−72	137.7	SGoM	N/A	N/A	Mexico	
120439 (F3)	MCP		−60	136.5	SGoM	N/A	N/A	Mexico	
120439 (F4)	MCP		−65	136.4	SGoM	N/A	N/A	Mexico	
	mean		−31.0	46.7		5058.3	2.4		10
	SD		20.3	41.0		4003.7	0.7		
	min		−72.0	0.6		48.8	1.0		
	max		−2.0	138.4		10108.0	4.0		

First foraging area for an individual turtle denoted as F1, followed by F2 (second foraging area), F3 (third foraging area), etc. A “1” in Oil Spill footprint column denotes that location of foraging centroid was within the surface oiling footprint of the Deepwater Horizon Oil Spill; see text for links to spatial layers. Foraging region abberviations are as in text. Jurisdiction: If state is listed, the centroid is within the State Submerged Lands (SSL) for that state. All SSL are within USA Exclusive Economic Zone (EEZ). The name of a country indicates the centroid is within that country’s EEZ.

*same turtle tracked/observed in 2011 and 2012.

**turtles from Hart et al (2012), refer to Methods S1 for details.

### Geographic regions

In this study, loggerheads selected foraging sites in each of the 5 regions defined earlier (Table 4). Turtles selected foraging sites in WFL most often (16/44 = 36%), and sites in NGoM second most frequently (14/44 = 32% Figure S7, see also Figure 3). Tagged loggerheads also selected foraging sites in Mexico, with 18% (8/44) of loggerheads in this study traveling to locations off the Yucatan Peninsula (SGoM). Fewer loggerheads selected foraging sites in SNWA (5/44 = 11%). Only 1 turtle selected a foraging site in WGoM (1/44 or 2%), and no turtles left the Gulf of Mexico. In a test of mean size of turtles by foraging site region, an ANOVA did not reveal significant difference (F_2,41_ = 0.071, p-value = 0.931; means: NGoM = 93.9 cm SCL-tip); SGulf (SGoM+SNWA)  = 94.6; WFL = 94.4). The results of the Chi-square test did not indicate that the proportion of turtles in each foraging site region was significantly different between turtles tagged in Florida and Alabama (χ^2^ = 2.25, p-value = 0.3246); however, this analysis was based on small number of turtles in each region.

### Foraging ‘hotspots’

**Figure 4:**
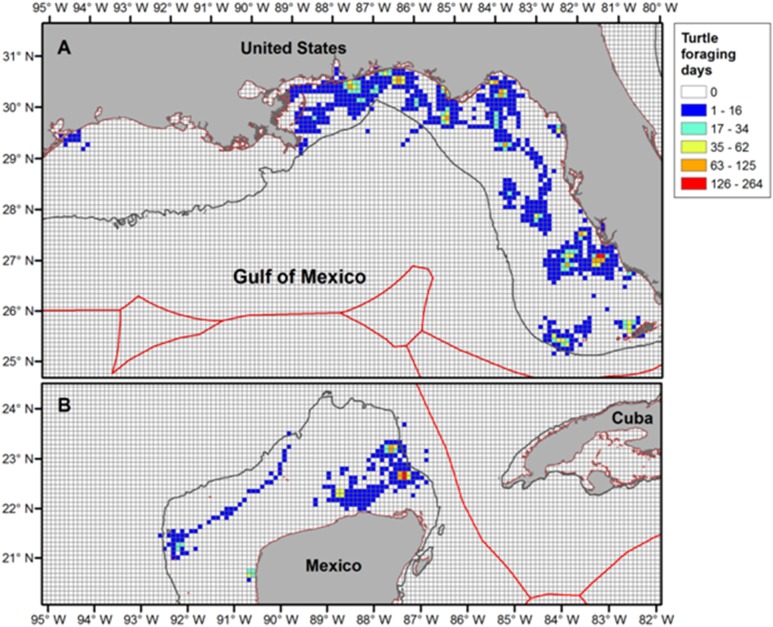
Foraging-days grid for Northern Gulf of Mexico (NGoM) loggerheads (*Caretta caretta*) in the U.S. Exclusive Economic Zone (EEZ; Panel A). Foraging-days grid for NGoM loggerheads in the Mexican EEZ. EEZ layers (Panel B; version 7, updated 20 November 2012) downloaded on 16 December 2013 from MarineRegions.org: http://www.marineregions.org/downloads.php.

**Figure 5:**
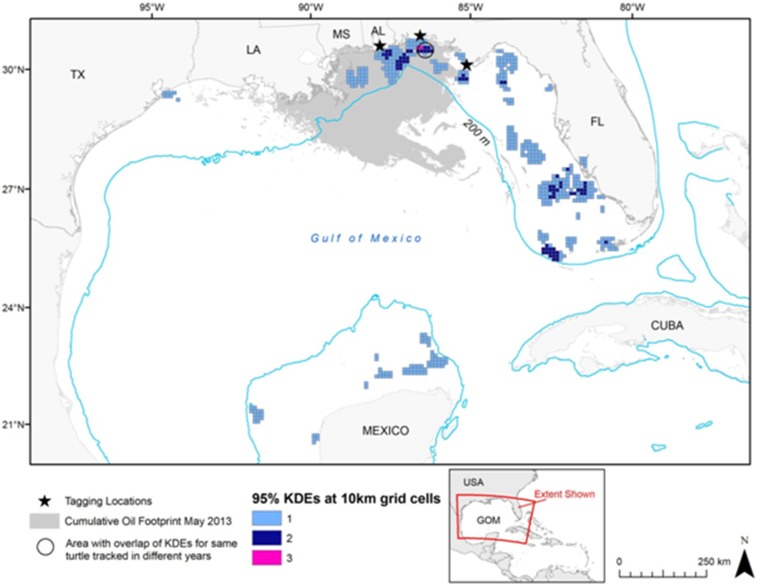
Number of loggerhead foraging home ranges (95% kernel density estimates) per 10 x10 km grid cell in the Gulf of Mexico.

High numbers of turtle-days per grid cell occurred during foraging at locations throughout the Gulf (Figure 4). The distribution of “hotspots” where foraging areas were concentrated included areas in NGoM, WGoM, and SGoM. A grid of home ranges (KDEs) per grid cell (Figure 5) also highlighted hotspots in more regions (NGoM, SWFL, SNWA, and SGoM) where multiple individuals displayed resident foraging behavior.

### Foraging areas and anthropogenic activities

**Figure 6:**
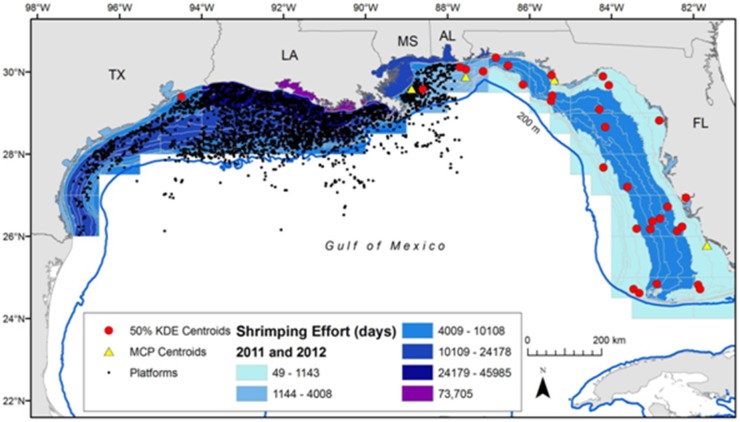
The 50% Kernel density estimates (KDE; circles) and Minimum Convex Polygons (MCP; triangles) centroids for 36 adult female loggerhead turtles (*Caretta caretta*). If a turtle had more than one centroid, only one is shown, with the KDE or MCP with the most points chosen and KDEs given priority. Centroids shown over shrimp trawling (days fished from 2011–2012) and oil and gas platform layers. Shrimp trawling data and statistical zone cutoffs provided by NOAA (Jim Nance and Amanda Frick, pers. comm.). The layer for oil and gas platforms was obtained from http://www.data.boem.gov/homepg/data_center/mapping/geographic_mapping.asp, accessed on 4 December 2013. Platforms in this layer included all platforms existing in the U.S. Bureau of Ocean and Energy Management (BOEM) database.

The spatial information for oil and gas platforms only extended into U.S. waters so we could only compare the locations of n = 37/45 centroids (82%, 36 turtles). These platforms currently do not spread into waters off the Florida coast and therefore many of these turtle centroids were greater than 10 km from a platform. Only 4 centroids were within 10 km of platforms and these centroids were located off the coasts of Alabama (n = 1), Mississippi/Louisiana (n = 2) and Texas (n = 1; Figure 6). Shrimp trawling information also covered U.S. waters. These turtle foraging area centroid locations (n = 37) fell within various subzones (divided by the NOAA statistical grid and by depth; Figure 6). The summed trawling effort (2011–2012, all seasons) for subzones with centroids ranged from 48.8–10108.0 days fished (mean ± SD = 5058.3±4003.7 days fished). Location of all U.S. foraging area centroids were within trawled waters (Figure 6); comparable layers for Mexico were not available. Further, 10 centroids overlapped with the surface oiling extent of the DWH oil spill (Figures 3 & 6). Additionally, a total of 2029 out of 5599 turtle foraging days (36.2%) overlapped with the spatial extent of this spill. Nine centroids were in State Submerged Lands (n = 8 in Florida and n = 1 in Texas); these 9 plus an additional 28 (n = 37) were within the U.S. EEZ and 13 centroids were within the Mexican EEZ.

## Discussion

For many marine species, locations of key foraging areas are beginning to emerge through the combined use of satellite tracking technologies and advanced spatial modeling approaches. Loggerheads in the NGoM subpopulation may be in decline [22], [23], thus documenting the location of foraging “hotspots” is critical for identifying potential at-sea threats that might warrant conservation action [18]. Results of this study highlight the year-round use of habitats in the GoM by loggerheads that nest in the Northern Gulf (Table 2 lists foraging dates), and this information can be used in efforts to designate critical in-water habitat for this subpopulation. Moreover, our findings indicate that foraging areas of NGoM loggerheads and anthropogenic activities overlap and are found in both the U.S. and Mexico. Thus, protection of females in this subpopulation requires both international collaborations and management of threats in these spatially overlapping areas.

Using the SSM technique, we were able to quantitatively identify the initiation and termination of the migratory period, as well as date of arrival of loggerheads at foraging grounds. Previous studies have defined departure from nesting beaches or arrival at foraging grounds through visual inspection of location data [1], [14], [15], [73], [74] or use of travel speed differences and absence of overlap in home range estimates [75]. The SSM technique provides a repeatable, robust method of quantitatively identifying changes in behavioral mode, so time at foraging grounds or in migration mode can be more accurately quantified.

### Migration periods

For loggerheads in the Northern Gulf, timing of peak post-nesting migration clearly varied by individual and year, supporting previous findings of Rees et al. [74] that those with foraging sites in different geographic regions may begin post-nesting migrations at different times of the nesting season. Although it is not yet known what drives this timing of migration, the timing of nesting activity may differ among years depending upon sea surface temperature (SST) [76], [77]. Our results highlight the time period in which post-nesting migrations for NGoM loggerheads is concentrated, and managers can use this information to prioritize time-dependent strategies that may protect migrating loggerheads such as has been done for the right whale (*Eubalaena glacialis*) along the U.S. Atlantic coast [78]. In that case, Seasonal Management Areas (SMA) were established to protect migrating right whales from shipping activities [78]–[80]. Similar regulations have been put in place off the Southern California coast to protect a variety of migrating whales from ships entering and leaving the port of Los Angeles [81]. As it has been suggested that migrating turtles travel predominately near the surface and that vessel-strike injuries appear most predominate in adult turtles [14], the risk from shipping activities could be significant [82].

One other study (see [15]) tracked a post-nesting loggerhead tagged in the NGoM to the WGoM. Although we observed only one clear migratory track going west-ward around Louisiana to Texas waters, we also obtained intermittent, and presumably migration, locations for turtle 108171 near Padre Island, Texas; this turtle (108171) ultimately settled at a foraging area off the Yucatan Peninsula, Mexico, however, her sporadic path was not across deep water directly to the Yucatan (unlike 2 of the NGoM turtles in Hart et al. [2]). Additional tracking of loggerheads in the NGoM subpopulation would be valuable to further delineate migratory corridors for loggerheads in the GoM, and better quantify the overlap of their migration paths with specific regional threats. For example, as described in Foley et al. [15], interactions with the GoM reef fish fishery are a serious concern for turtles selecting foraging sites in the WFL region.

### Foraging area characteristics

In our study, the mean size of core foraging areas for loggerheads tagged in the NGoM was 100.7 km^2^, similar to findings presented in Hart et al. [2] and those presented in Zbinden et al. [83] (Mediterranean), Marcovaldi et al. [1] (Brazil), Foley et al. [15] (U.S.), and to neritic open-sea (over 2 km from shore in water less than 200 m deep) loggerheads from Schofield et al. [84] (Mediterranean). As in these other studies and Tucker et al. [85], turtles from our study exhibited significant fidelity to their core foraging area. In fact, one turtle tracked twice during the study (turtle #106345/119944) migrated to the same distinct foraging site in successive years, with centroids of her 2011 and 2012 50% KDEs separated by only 1.1 km and a difference in size of her 50% KDEs of only 4.3 km^2^. This selection of almost the exact same ‘patch’ of foraging habitat by a loggerhead is similar to findings presented in Marcovaldi et al. [1] on repeated use of remarkably similar individual foraging sites for Brazilian loggerheads tracked >1200 days, and findings of one turtle in Foley et al. [15] that had 91% of its primary residence area in 2001 within that same area it occupied in 2000. And 10 years of data from Schofield et al [86] that showed over 100 loggerheads primarily used a small area only 7 km×1 km in the Mediterranean. Documentation of site residency at discrete foraging areas for loggerheads has grown in recent years at various study sites around the globe, using a range of methods ([87] Australia, mark-recapture; [1] Brazil, satellite tracking; [7] Atlantic coast of U.S., stable isotopes). In our study, we used quantitative site fidelity tests to document long-term foraging site-residency where individuals displayed resident behavior (see also Figure S4). This site-loyalty was further demonstrated through our addition of tracking data during hundreds of additional tracking days for 2 turtles previously summarized in Hart et al. [2]. Specifically, we did not see a large change in 50% KDE size for these 2 turtles (see Figure S4), despite KDE analysis that included an additional 551 combined foraging days; this result supports the finding of Foley et al. [15] that after 100 days of tracking at a foraging ground, further increases in foraging area size were minimal. Such local residency implies that forage resources located within loggerhead core-use areas are exploited by turtles throughout tracking durations. This consistency in size of core-use areas in the GoM (see [15]) also indicates that perturbations at foraging sites could be monitored using satellite- and GPS-tracking techniques; we would expect turtles to establish core-use areas of approximately 100 km^2^ and home ranges of approximately 500 km^2^ (see [15]). However, currently little is known about the condition and quality of resources available at most of these foraging areas. Coarse characterization of predominantly WFL shelf loggerheads in Foley et al. [15] provided information on dominant sediment type, however, focused studies of benthic composition and prey availability at known foraging sites would be an important next step in further understanding foraging habitat composition.

Several previous studies described variability in foraging strategies of post-nesting loggerheads [74], [88]–[90]. Hatase et al. [88] and Hawkes et al. [89] suggested that off Japan and Cape Verde, larger individuals foraged in more productive neritic waters whereas smaller individuals foraged in oceanic (i.e., deeper) waters. However, off Oman [74] and in the GoM [2], [90], there was little correlation between body size and water depth at foraging sites. In our study, all turtles, regardless of size, foraged in neritic waters ranging from −2 to −72 m deep. We found no relationship between size and any variables including water depth, migration distance or size of the core-use foraging area. This finding is in opposition to what Foley et al. [14] recently reported for 14 turtles tracked earlier in the NGoM. In their study, larger turtles migrated further and took less direct routes during migration than smaller turtles. We also found no significant difference in size of turtles among foraging locations which is in opposition to recent findings by Vander Zanden et al. [7] who reported differences in body size for turtles nesting on the U.S. Georgia coast that used 3 distinct foraging sites. These different results for our study versus those reported in Foley et al. [14] may simply reflect variations in sample size between the two studies; we tracked 59 turtles whereas Foley et al. [14] tracked 14 from the NGoM subpopulation. However the lack of correlation we found between body size and foraging location, as reported by Vander Zanden et al. [7], suggests that in the GoM, body size may not be a predictor of foraging site location. This may be because turtles nesting in the northern Gulf remain in the GoM; therefore locations of foraging areas for these turtles could vary by only ∼5° latitude whereas foraging areas for turtles reported by Vander Zanden et al. [7] varied by ∼17° latitude. The lack of wide temperature variation among GoM foraging sites may result in similar habitat quality or resource availability for all turtles. Thus, our results confirm that not all loggerhead populations exhibit phenotypic dichotomies in movement patterns or selection of foraging sites.

Loggerhead use of secondary foraging sites has been observed for several other studies, most recently documented as 20% of turtles tracked in Foley et al. [15] and 13% of those tracked in Griffin et al. [16]. For the 44 NGoM loggerheads in our study that were tracked to distinct foraging sites, we observed only 4/44 (9%) of turtles had secondary or additional foraging site(s). The difference in proportions for our study versus Foley et al. [15] may be attributed to the fact that turtles from 3 rookeries (nesting subpopulations) were included in Foley et al. [15] whereas our focus was the NGoM subpopulation. The 4 turtles in our study that selected secondary or additional foraging sites did so in 3/5 of the geographic regions: NGoM (turtle 119942), WFL (turtle 52968), and SGoM (turtles 120439 and 129296). Depths of foraging site centroids for these turtles were all <−72 m and distances between successive foraging site centroids were all <5.6 km (see Table 4). Thus, we did not observe that loggerheads residing at lower latitudes remained at a single foraging site and those at higher latitudes had additional winter or secondary foraging sites; latitude and depth in our study did not appear to be the main determinants of whether loggerheads used additional foraging sites, as was suggested by Foley et al. [15]. With a mean tracking period at foraging sites of 133.5 days (∼4 months) in our study, it is possible that other turtles in our study selected secondary or additional foraging sites after tracking periods ceased. Longer tracking periods of additional individual turtles at foraging sites would help to resolve trends in NGoM loggerhead use of 1 or more foraging sites in the GoM.

### Geographic regions

Although foraging grounds for loggerheads have been documented hundreds to thousands of kilometers from their nesting beaches [1], [2], [13], [15], [16], loggerheads nesting in the NGoM appear to forage exclusively in the GoM. Most (69%) of the turtles in this study followed the Type A1 post-nesting movement pattern as described by [91] by making oceanic or coastal movements to a neritic foraging ground. However the remaining 31% followed the A3 pattern and remained as residents near their nesting beaches. Loggerheads in the Mediterranean have also been shown to forage predominately in that ocean basin [91]. These findings underscore the importance of GoM habitats for loggerheads in this subpopulation, and the fact that anthropogenic threats in the GoM (see below) will impact not only their nesting habitat but also their inter-nesting areas [26] and foraging habitats (*this study* and [2]).

Our regional rankings of foraging sites indicate that the greatest proportion of turtles in this subpopulation forage in the WFL region (36%), followed by NGoM (32%), SGoM (18%), SNWA (11%), and WGoM (2%). Of the turtles selecting foraging sites in the NGoM, 35% were tagged in Alabama and 14% were tagged in Florida. These rankings mirror those found in Foley et al. [14], [15] with a smaller data set, and different turtles than those tracked here (A. Foley and M. Nichols, pers. comm.). The regional rankings also support results in other studies that showed heavy use of the WFL coast by foraging turtles [85], [90]. Further, our results provide further empirical evidence in support of recent critical habitat designations for the species [92]. However, our findings also indicate that the extent of critical in-water habitat would need to be expanded to include the zones we have identified here as “high use” areas (see Figures 4 and 5). The currently-proposed designation defines critical areas in the NGoM for breeding only, whereas our findings, and those of Hart et al [33], illustrate their added importance as key foraging and inter-nesting habitats. In addition, the proposed designation of in-water critical foraging habitat only includes habitat from Mean High Water to 1.6 km offshore whereas our study shows mean distance of core use foraging areas from shore for all turtles was 46.7 km and for turtles foraging in NGoM core areas was 24.7 km. Hart et al. [33] also showed this area provides important inter-nesting habitat with inter-nesting home ranges (50% KDEs) located a mean distance of 33 km from shore. These findings suggest critical habitat designations for these loggerheads should extend farther beyond the shoreline and include not only breeding habitat [33], but also foraging hotspots and migratory corridors.

Even though we tracked a large number of turtles in this study, the map of foraging site locations for this subpopulation may still be incomplete. In a two decade-long study on European shags (*Phalacrocorax aristotelis*) [93], researchers found that foraging distribution over the entire study period was concentrated in 3 areas. However, data from year 1 and 2 captured an average of 54% and 64% of this distribution, respectively, but it required 8 years of data to capture more than 90% of the entire distribution. In our study, we discovered additional foraging areas with each additional year of the tracking data from 2010–2013, supporting findings of Schofield et al. 2013 [94] that promote larger sample sizes for more complete mapping of foraging locations. Given recent estimates of remigration intervals of 3.4, 4.1, and 5.0 years for loggerheads nesting at Wassaw Island, Georgia, U.S. and going to 3 different foraging areas in the Atlantic [7], and 3.2 years for loggerheads nesting at Keewaydin Island, Florida [95], additional tracking of loggerheads from our tagging sites would be valuable to complete the picture of overall distribution of foraging sites for this loggerhead subpopulation. For example, in our last year of the study (2013), we mapped a “new” foraging site location for our study in the WGoM, near Houston, Texas (see Figure 3). Even though locations in Foley et al. [15] and Hart et al. [2] are similar to this study, the appearance of new foraging sites on the overall map suggests that there may still be foraging areas yet unidentified. As well, the proportion of turtles traveling to different foraging regions differed annually and between study sites. Future predictive habitat modeling efforts that use values presented here and in Foley et al. [15] would be valuable for identifying other possible foraging habitat for loggerheads in the GoM. In addition, turtles that we tagged after nesting in Alabama used 5 different foraging areas whereas turtles tagged after nesting in Florida used only 3 foraging grounds, and a larger proportion of turtles tracked from Florida than from Alabama traveled to Mexico. Thus, we suggest that continued tracking of turtles from these, and other, nesting beaches in the NGoM is warranted. Additional complementary studies using analysis of loggerhead stable isotope ‘signatures’ would also be extremely valuable for further characterizing the proportion of turtles within the overall NGoM subpopulation that use different foraging regions [7] and for confirming whether discrete site-selection is repeatable, as we observed with the two tracking periods for turtle 106345/119944 in 2011 and 2012, respectively.

In our study, nearly 25% of tracked loggerheads traveled to and/or foraged in international waters off either Mexico or Cuba, part of the SGoM region; this result reinforces the need for international cooperation in the conservation and recovery of this species [2], [15], [18]. The use of Cuban waters as a migratory corridor and/or foraging area is a particularly important conservation concern as there is documentation of fishermen in this area taking post-nesting loggerheads [90], [96], [97]; 3 of our tracks also ceased immediately for turtles there on previously ‘normal’ migrations (see Figure 3). A complete ban on the harvest of all marine turtles in Cuba was instituted in January 2008, however, tracking results in this and other studies [98] suggest further investigation into the continued harvest of several species of marine turtles in Cuban waters may be warranted.

### Foraging ‘hotspots’

Loggerheads in near-shore NGoM waters may be exposed to incidental capture in shrimp trawls, oil spills, dredging, hypoxia (i.e., low levels of dissolved oxygen in bottom waters), and other threats. Although foraging habitat characteristics and suitability for marine turtles in this region are poorly understood, locations of core-use foraging habitats identified here (e.g., after the DWH oil spill) indicate that important habitat exists for loggerheads at these same potentially affected sites [99]. This is also the case for endangered Kemp’s ridleys [12]. Whether such at-sea foraging sites previously used by loggerheads will continue to be used with equal frequency in the future, or alternatively abandoned, remains to be seen; it is possible that environmental conditions at some of these sites in the NGoM have been altered by large-scale perturbations such as the DWH oil spill [12], [35], [99] and “dead zones” of hypoxic conditions [100], [101].

With the help of satellite telemetry and other techniques (see [102]), the locations of in-water foraging areas for marine turtle species throughout the world are becoming better defined [2], [12]–[16], [94], [67], [103]. In the GoM, Shaver et al. [12] found that post-nesting adult female Kemps ridleys primarily foraged in neritic habitat off Louisiana, along the West Florida shelf, and off the tip of the Yucatan Peninsula, Mexico. It appears that the areas identified by Shaver et al. [12] overlap with a proportion of the foraging sites we identified for loggerheads (see Figure 4); this result indicates that there are areas in the NGoM, in particular, that serve as important foraging habitat for 2 marine turtle species. It is possible that these same areas may also be important marine habitat for Gulf sturgeon (*Acipenser oxyrinchus desoto*) [104], West Indian manatees (*Trichechus manatus*) [105], and bottle-nosed dolphins (*Tursiops truncatus*) [106]. Whether the areas we identified here and those in Shaver et al. [12] also serve as critical habitat for male marine turtles in the GoM is unknown, and this is an obvious gap in our knowledge (but see [107], [108], [94]).

### Foraging areas and anthropogenic activities

Many turtle home ranges overlapped with areas heavily used by commercial trawlers (see Figure 5). In their comparison of shrimping effort versus turtle density in the GoM, McDaniel et al. [32] previously indicated that neritic waters off of our study sites supported ‘medium’ shrimping effort but ‘low to medium’ turtle density; regardless, our results suggest a higher degree of overlap with shrimping and core-use loggerhead foraging areas than previously recognized.

Fewer home ranges overlapped with areas with active oil and gas platforms, as the majority of the turtles we tracked foraged off WFL and the NGoM, where few oil and gas platforms are located. However, our SSM results indicated that neritic habitat off Texas, Louisiana, Mississippi, and Alabama, where oil and gas platforms are prevalent, appears to serve both as important movement corridors and foraging sites for turtles nesting throughout the NGoM (see also [33]). Oil and gas platforms are often characterized as important oceanic microcosms for GoM biota [109], [110], and loggerhead use of underwater supports of these structures has been documented [111]. In spite of this, activities associated with these platforms may pose a risk of injury to marine turtles, as active drilling rigs and platforms require service and supply activities that increase boat traffic in their vicinity, while the construction and demolition of these rigs may serve as further hazards to marine turtles inhabiting or migrating through the vicinity [112]. In addition, 10 of our loggerhead foraging area centroids fell within the ‘footprint’ of surface oiling extent of the DWH oil spill (Figure 3), with 36.2% of the foraging days (2029/5599) located within this surface oiling footprint. Thus, the extent of adult female loggerhead interaction with active trawling and oil and gas extraction activities may require further evaluation; our results have direct implications for DWH oil spill exposure modeling for loggerheads that use the NGoM, and in particular for those that remain in the NGoM at foraging sites.

Due to the varied anthropogenic threats in the area, a cumulative impact assessment in the GoM could inform management efforts. A 2008 global impact assessment on marine ecosystems found the GoM to be an area with a medium-high impact from human activities [113], and we propose that analyses of tracking data combined with the spatial extent of known threats can contribute to effective management of marine resources (see [114]).

## Conclusions

Our results highlight the importance of habitats within the GoM for sustaining nesting females within the NGoM loggerhead subpopulation, and they clearly demonstrate the potential for interactions with anthropogenic activities that may threaten population persistence and recovery. Because adult female survival rates have an especially strong effect on population recovery [115], [116], designing management strategies to protect adult females is essential.

The DWH oil spill is a key concern for exposure of adult females in this declining subpopulation to oil and oil clean-up activities. Potential impacts of oil and dispersants from the DWH oil spill on Gulf sea turtles may range from mortality to sublethal stress and chronic impairment, including potential deleterious effects on reproduction and recruitment [34]. Sublethal or latent effects, such as harm to the lungs, would not be detectable by physical examination. Foraging turtles may also be subject to continued exposure and adverse effects of oil, dispersant, and associated chemicals that persist in the marine environment, including in the marine food web. Indirect impacts from potential habitat degradation and loss of prey resources may further reduce sea turtle survival and reproduction. Because the DWH because the DWH oil spill occurred in deep, offshore waters, that spill was an event that may have impacted species at risk by breaking the food chain links to particle feeders and thus to even higher trophic levels [99]. Our finding that 32% of the loggerheads in our study remained in and took up residence at sites in NGoM foraging habitats year-round indicates that an environmental perturbation, such as an oil spill, may have more far-reaching effects than previously believed, as adult female loggerheads may be impacted not only during nesting and inter-nesting periods [33], but also during migration and at the foraging grounds identified here. Analyses of long-term loggerhead capture-recapture records, like in Phillips et al. [95] will be extremely valuable for understanding survival rates of individuals in this subpopulation and how they compare to vital rates of other loggerhead subpopulations in the Northwest Atlantic.

## Supporting Information

Figure S1 **Predicted and actual tracks for Turtles 119941, 129506 and 129515 to show examples of exceptions for migration days (migration periods not directly before foraging periods, see Methods S1).** Turtle 119941 had a small stop-over at-sea (blue points at-sea off NW Florida coast) in between migration (red points), and both the migration before and after were used. Turtle 129506 had two foraging periods as defined by our date cut-off (all blue points). The first was in the inter-nesting area near land and this period did not pass site fidelity. The visual inspection confirmed a ‘main’ migration after this first foraging period (red points). Turtle 129515 had a ‘main’ migration (red points) before an early small foraging period that was not the main foraging period used for analysis (blue points near St. Joseph Peninsula amidst other blue points used as foraging area. Yellow points in this area represent the small migration directly preceding the main foraging time).
(TIF)

Figure S2 **Migration tracks for 46 adult female loggerheads (** ***Caretta caretta*** **) after nesting in the Northern Gulf of Mexico 2010–2013.** Tagging locations (black stars) from left to right are Gulf Shores, AL, Eglin Air Force Base, FL and St. Joseph Peninsula, FL. Lines created by connecting main migration locations (see Methods S1) filtered by swim speed with erroneous locations (land or very distant) removed.
(TIF)

Figure S3 **Example switching state-space model (SSM) prediction (red and blue points) over raw unfiltered locations (open grey circles) for Turtle119946 tagged in Gulf Shores, Alabama in 2012.**
(TIF)

Figure S4 **Foraging site kernel density estimate (KDE; 50%) for 2 adult female loggerheads (** ***Caretta caretta*** **) tagged in 2010 and previously summarized in Hart et al. (2012), shown in dark blue, and with an additional 489 days (turtle 52968) and 62 days (turtle 47755) of tracking data added during ‘foraging’ mode (light blue).**
(TIF)

Figure S5 **Foraging site kernel density estimates (KDE; 95% and 50%) for adult female loggerheads (** ***Caretta caretta*** **) that nested in the Northern Gulf at study sites in Alabama and Florida between 2010–2013.** Red asterisks denote KDEs for 2 turtles from previous study (Hart et al. 2012).
(TIF)

Figure S6 **Ten minimum convex polygon (MCP) areas for 7 adult female loggerheads (** ***Caretta caretta*** **) satellite-tagged in the Northern Gulf (Turtle 129496 had 3 MCPs and Turtle 120439 had 2). MCPs are colored by tagging location.**
(TIF)

Figure S7 **Kernel Density Estimation (KDE) for 38 adult female loggerheads (40 KDEs;** ***Caretta caretta*** **) satellite-tagged in the Northern Gulf of Mexico (NGoM); contours are colored by original tagging location (GS = Gulf Shores, SJP = St. Joseph Peninsula, EAFB = Eglin Air Force Base).** One turtle was tracked in both 2011 and 2012, and turtle 120439 had two foraging KDEs. Two turtles from Hart et al. (2012) are included. Gulf regions are denoted by red lines and are as follows: WGoM, Western Gulf of Mexico; NGoM; WFL, Western Florida; SNWA, Subtropical Northwest Atlantic; and SGoM, Southern Gulf of Mexico.
(TIF)

Table S1 **Turtle info.**
(DOCX)

Table S2 **Tags used by site and year.**
(DOCX)

Table S3 **SSM prediction and model parameters for turtles 119946, 129515, 119941, and 129506.**
(DOC)

Methods S1 **Detailed methods for why we selected or ruled out turtles for specific analyses and any exceptions made.**
(DOCX)

## References

[1] Marcovaldi MÂ, Lopez GG, Soares LS, Lima EHSM, Thomé JCA, et al (2010) Satellite tracking of female loggerhead turtles highlights fidelity behavior in northeastern Brazil. Endanger Species Res 12: 263–272 10.3354/esr00308

[2] Hart KM, Lamont MM, Fujisaki I, Tucker AD, Carthy RR (2012) Common coastal foraging areas for loggerheads in the Gulf of Mexico: Opportunities for marine conservation. Biol Conserv 145: 185–194 10.1016/j.biocon.2011.10.030

[3] Hays GC, Scott R (2013) Global patterns for upper ceilings on migration distance in sea turtles and comparisons with fish, birds and mammals. Funct Ecol 27(3): 748–756 10.1111/1365-2435.12073

[4] Hamann M, Limpus C, Whittier J (2002) Patterns of lipid storage and mobilisation in the female green sea turtle (*Chelonia mydas*). J Comp Physiol B 172(6): 485–493 10.1002/s00360-002-0271-2 12192510

[5] Zbinden JA, Bearhop S, Bradshaw P, Gill B, Margaritoulis D, et al (2011) Migratory dichotomy and associated phenotypic variation in marine turtles revealed by satellite tracking and stable isotope analysis. Mar Ecol Prog Ser 421: 291–302 10.3354/meps0887

[6] Hatase H, Omuta K, Tsukamoto K (2013) A mechanism that maintains alternative life histories in a loggerhead sea turtle population. Ecology 94: 2583–2594 10.1890/12-1588.1 24400510

[7] VanderZanden HB, Pfaller JB, Reich KJ, Pajuelo M, Bolten AB, et al (2014) Foraging areas differentially affect reproductive output and interpretation of trends in abundance of loggerhead turtles. Mar Biol: 1–14. 10.1007/s00227-013-2361-y

[8] National Marine Fisheries Service and U.S. Fish and Wildlife Service (2008) Recovery plan for the Northwest Atlantic population of loggerhead sea turtle (*Caretta caretta*), second revision. National Marine Fisheries Service, Silver Spring, MD.

[9] Block BA, Jonsen ID, Jorgensen SJ, Winship AJ, Shaffer SA, et al (2011) Tracking apex marine predator movements in a dynamic ocean. Nature 475(7354): 86–90 10.1038/nature10082 21697831

[10] Fossette S, Witt MJ, Miller P, Nalovic MA, Albareda D, et al (2014) Pan-Atlantic analysis of the overlap of a highly migratory species, the leatherback turtle, with pelagic longline fisheries. Proc R Soc B 281: 20133065 10.1098/rspb.2013.3065 PMC4027393 24523271

[11] Scott R, Hodgson DJ, Witt MJ, Coyne MS, Adnyana W, et al (2012) Global analysis of satellite tracking data shows that adult green turtles are significantly aggregated in Marine Protected Areas. Global Ecol Biogeogr 21(11): 1053–1061 10.1111/j.1466-8238.2011.00757.x

[12] Shaver DJ, Hart KM, Fujisaki I, Rubio C, Sartain AR, et al (2013) Foraging area fidelity for Kemp’s ridleys in the Gulf of Mexico. Ecol Evol 3(7): 2002–2012 10.1002/ece3.594 23919146 PMC3728941

[13] Hawkes LA, Witt MJ, Broderick AC, Coker JW, Coyne MS, et al (2011) Home on the range: spatial ecology of loggerhead turtles in Atlantic waters of the USA. Divers Distrib 17: 624–640. 10.1111/j.1472-4642.2011.00768.x

[14] Foley AM, Schroeder BA, Hardy R, MacPherson SL, Nicholas M, et al (2013) Postnesting migratory behavior of loggerhead sea turtles *Caretta caretta* from three Florida rookeries. Endanger Species Res 21: 129–142 10.3354/esr00512

[15] Foley AM, Schroeder BA, Hardy R, MacPherson SL, Nichols M (2014) Long-term behavior at foraging sites of adult female loggerhead sea turtles (*Caretta caretta*) from three Florida rookeries. Mar Biol. 10.1007/s00227-014-2415-9 PMC4033788 24882883

[16] Griffin DB, Murphy SR, Frick MG, Broderick AC, Coker JW, et al (2013) Foraging habitats and migration corridors utilized by a recovering subpopulation of adult female loggerhead sea turtles: implications for conservation. Mar Biol 160(12): 3071–3086 10.1007/s00227-013-2296-3

[17] Turtle Expert Working Group (2009) An assessment of the Loggerhead Turtle Population in the Western Northern Atlantic Ocean. 131 p. NOAA Technical Memorandum NMFS-SEFSC-575. DOI:10.1007/s00227-005-0076-4.

[18] Hamann M, Godfrey MH, Seminoff JA, Arthur K, Barata PCR, et al (2010) Global research priorities for sea turtles: informing management and conservation in the 21st century. Endanger Species Res 11: 245–269 10.3354/esr00279

[19] Shamblin BM, Dodd MG, Bagley DA, Ehrhart LM, Tucker AD, et al (2011) Genetic structure of the southeastern United States loggerhead turtle nesting aggregation: evidence of additional structure within the peninsular Florida recovery unit. Mar Biol 158: 571–587 10.1007/s00227-010-1582-6

[20] Shamblin BM, Bolten AB, Bjorndal KA, Dutton PH, Nielsen JT, et al (2012) Expanded mitochondrial control region sequences increase resolution of stock structure among North Atlantic loggerhead turtle rookeries. Mar Ecol-Prog Ser 469: 145–160 10.3354/Meps09980

[21] Richards PM, Epperly SP, Heppell SS, King RT, Sasso CR, et al (2011) Sea turtle population estimates incorporating uncertainty: a new approach applied to western North Atlantic loggerheads (*Caretta caretta*). Endanger Species Res 15: 151–158 10.3354/esr00379

[22] Witherington B, Kubilis P, Brost B, Meylan A (2009) Decreasing annual nest counts in a globally important loggerhead sea turtle population. Ecol Appl 19: 30–54 10.1890/08-0434.1 19323172

[23] Lamont MM, Carthy RR, Fujisaki I (2012) Declining reproductive parameters highlight conservation needs for loggerhead turtles (*Caretta caretta*) in the northern Gulf of Mexico. Chelonian Conserv Biol 11: 190–196 DOI 10.2744/CCB-1006.1.

[24] Lewison RL, Freeman SA, Crowder LB (2004) Quantifying the effects of fisheries on threatened species: the impact of the pelagic longlines on loggerhead and leatherback sea turtles. Ecol Lett 7: 221–231.

[25] Lewison RL, Crowder LB (2007) Putting longline bycatch of sea turtles into perspective. Conserv Biol 21(1): 79–86. 17298513 10.1111/j.1523-1739.2006.00592.x

[26] Finkbeiner EM, Wallace BP, Moore JE, Lewison RL, Crowder LB, et al (2011) Cumulative estimates of sea turtle bycatch and mortality in USA fisheries between 1990 and 2007. Biol Conserv 144: 2719–2727.

[27] Jennings S, Kaiser MJ (1998) The effects of fishing on marine ecosystems. Adv Mar Biol 34: 201–352 10.1016/S0065-2881(08)60212-6

[28] Schwinghamer P, Gordon DC, Rowell TW, Prena J, Keown DLMC, et al (1998) Effects of experimental otter trawling on surficial sediment properties of a sandy-bottom ecosystem on the Grand Banks of Newfoundland. Conserv Biol 12: 1215–1222 10.1046/j.1523-1739.1998.0120061215.x

[29] Hansson M, Lindegarth M, Valentinsson D, Ulmestrand M (2000) Effects of shrimp trawling on abundance of benthic macrofauna in Gullmarsfjorden, Sweden. Mar Ecol-Prog Ser 198: 191–201 10.3354/Meps198191

[30] Bolten AB (2003) Active swimmers-passive drifters: the oceanic juvenile stage of loggerheads in the Atlantic system. In: Bolten A, Witherington B, editors. Loggerhead sea turtles. Smithsonian Institution Press, Washington, 63–98.

[31] National Marine Fisheries Service (2002) ESA biological opinion on shrimp trawling in the southeastern United States under the Sea Turtle Conservation Regulations. NOAA NMFS, Washington, D.C., U.S.

[32] McDaniel CJ, Crowder LB, Priddy JA (2000) Spatial dynamics of sea turtle abundance and shrimping intensity in the U.S. Gulf of Mexico. Conserv Ecol 4(1): 15.

[33] Hart KM, Lamont MM, Sartain AR, Fujisaki I, Stephens BS (2013) Movements and habitat-use of loggerhead sea turtles in the Northern Gulf of Mexico during the reproductive period. PLoS ONE 8(7): e66921 10.3354/esr00379 23843971 PMC3700946

[34] Bjorndal KA, Bower BW, Chaloupka M, Crowder LB, Heppell SS, et al (2011) Better science needed for restoration in the Gulf of Mexico. Science 331: 537–538 10.1126/science.1199935 21292956

[35] Campagna C, Short FT, Polidoro BA, McManus R, Collette BB, et al (2011) Gulf of Mexico oil blowout increases risks to globally threatened species. Bioscience 61: 393–397 10.1525/bio.2011.61.5.8

[36] National Marine Fisheries Service Southeast Fisheries Science Center (2008) Sea turtle research techniques manual. 92 p. NOAA Technical Memorandum NMFS-SEFSC-579.

[37] Jones TT, Van Houtan KS, Bostrom BL, Ostafichuk P, Mikkelsen J, et al (2013) Calculating the ecological impacts of animal-borne instruments on aquatic organisms. Methods Ecol Evol 4(12): 1178–1186 10.1111/2041-210X.12109

[38] Coyne MS, Godley BJ (2005) Satellite Tracking and Analysis Tool (STAT): an integrated system for archiving, analyzing and mapping animal tracking data. Mar Ecol-Prog Ser 301: 1–7.

[39] CLS (2011) Argos user’s manual: worldwide tracking and environmental monitoring by satellite. 19 August 2011 update. CLS, Toulouse. Available: http://www.argossystem.org/web/en/76-user-s-manual.php. Accessed 2013 Jun 9.

[40] Hays GC, Åkesson S, Godley BJ, Luschi P, Santidrian P (2001) The implications of location accuracy for the interpretation of satellite-tracking data. Anim Behav 61(5): 1035–1040 10.1006/anbe.2001.1685

[41] Witt MJ, Åkesson S, Broderick AC, Coyne MS, Ellick J, et al (2010) Assessing accuracy and utility of satellite-tracking data using Argos-linked Fastloc-GPS. Anim Behav 80(3): 571–581 10.1016/j.anbehav.2010.05.022

[42] Kalman RE (1960) A new approach to linear filtering and prediction problems. J Basic Eng 82(1): 35–45.

[43] Lopez R, Malardé JP (2011) Improving Argos Doppler location using Kalman filtering. Ramonville Saint-Agne, France.

[44] Jonsen ID, Myers RA, James MC (2006) Robust hierarchical state-space models reveal diel variation in travel rates of migrating leatherback turtles. J Anim Ecol 75: 1046–1057 10.1111/j.1365-2656.2006.01129.x 16922840

[45] Patterson TA, Thomas L, Wilcox C, Ovaskainen O, Matthiopoulos J (2008) State-space models of individual animal movement. Trends Ecol Evol 23(2): 87–94 10.1016/j.tree.2007.10.009 18191283

[46] Jonsen ID, Myers RA, Flemming JM (2003) Meta-analysis of animal movement using state-space models. Ecology 84: 3055–3063 10.1890/02-0670

[47] Jonsen ID, Flemming JM, Myers RA (2005) Robust state-space modeling of animal movement data. Ecology 86: 2874–2880 10.1890/04-1852

[48] Breed GA, Jonsen ID, Myers RA, Bowen WD, Leonard ML (2009) Sex-specific, seasonal foraging tactics of adult grey seals (*Halichoerus grypus*) revealed by state-space analysis. Ecology 90(11): 3209–3221 10.1890/07-1483.1 19967876

[49] Bailey H, Mate BR, Palacios DM, Irvine L, Bograd SJ, et al (2009) Behavioural estimation of blue whale movements in the Northeast Pacific from state-space model analysis of satellite tracks. Endanger Species Res 10: 93–106.

[50] Jonsen ID, Myers RA, James MC (2007) Identifying leatherback turtle foraging behaviour from satellite telemetry using a switching state-space model. Mar Ecol-Prog Ser 337: 255–264 10.3354/meps337255

[51] Bailey H, Shillinger G, Palacios D, Bograd S, Spotila J, et al (2008) Identifying and comparing phases of movement by leatherback turtles using state-space models. J Exp Mar Biol Ecol 356: 128–135 10.1016/j.jembe.2007.12.020

[52] Shillinger GL, Swithenbank AM, Bograd SJ, Bailey H, Castelton MR, et al (2010) Identification of high-use internesting habitats for eastern Pacific leatherback turtles: role of the environment and implications for conservation. Endanger Species Res 10: 215–232 10.3354/esr00251

[53] Benson SR, Eguchi T, Foley DG, Forney KA, Bailey H, et al (2011) Large-scale movements and high-use areas of western Pacific leatherback turtles (*Dermochelys coriacea*). Ecosphere 2(7): art84 10.1890/Es11-00053.1

[54] Maxwell SM, Breed GA, Nickel BA, Makanga-Bahouna J, Pemo-Makaya E, et al (2011) Using satellite tracking to optimize protection of long-lived marine species: olive ridley sea turtle conservation in Central Africa. PLoS ONE 6(5): e19905 10.1371/journal.pone.0019905 21589942 PMC3092776

[55] Eckert SA, Moore JE, Dunn DC, van Buiten RS, Eckert KL, et al (2008) Modeling loggerhead turtle movement in the Mediterranean: Importance of body size and oceanography. Ecol Appl 18: 290–308. 18488597 10.1890/06-2107.1

[56] White GC, Garrott RA (1990) Analysis of wildlife radiotracking data. Academic Press, New York, NY.

[57] Worton BJ (1987) A review of models of home range for animal movement. Ecol Model 38: 277–298 10.1016/0304-3800(87)90101-3

[58] Worton BJ (1989) Kernel methods for estimating the utilization distribution in home-range studies. Ecology 70: 164–168 10.2307/1938423

[59] Rodgers AR, Carr AP, Smith L, Kie JG (2005) HRT: Home Range Tools for ArcGIS. Thunder Bay, Ontario, Canada: Ontario Ministry of Natural Resources, Centre for Northern Forest Ecosystem Research.

[60] Worton BJ (1995) Using Monte Carlo simulation to evaluate kernel-based home range estimators. J Wildl Manage 59(4): 794–800.

[61] Seaman DE, Powell RA (1996) An evaluation of the accuracy of kernel density estimators for home range analysis. Ecology 77: 2075–2085 10.2307/2265701

[62] Laver PN, Kelly MJ (2008) A critical review of home range studies. J Wildl Manage 72(1): 290–298 10.2193/2005-589

[63] Environmental Systems Research Institute (ESRI) (2007) ArcGIS 9.3 GIS. Redlands, CA.

[64] Hooge PN, Eichenlaub W, Hooge ER (2001) Animal movement 2.5. US Geological Survey, Alaska Biological Science Center, Anchorage, AK.

[65] Burt WH (1943) Territoriality and home range concepts as applied to mammals. J Mammal 24(3): 346–352.

[66] Mohr CO (1947) Table of equivalent populations of North American small mammals. American Midl Nat 3(1): 223–249.

[67] Arendt MD, Segars AL, Byrd JI, Boynton J, Schwenter JA, et al (2012) Migration, distribution, and diving behavior of adult male loggerhead sea turtles (*Caretta caretta*) following dispersal from a major breeding aggregation in the Western North Atlantic Mar Biol 159: 113–125. 10.1007/s00227-011-1826-0

[68] Pajuelo M, Bjorndal KA, Reich KJ, Arendt MD, Bolton AB (2012) Distribution of foraging habitats of male loggerhead turtles (*Caretta caretta*) as revealed by stable isotopes and satellite telemetry. Mar Biol 159(6): 1255–1267 10.1007/s00227-012-1906-9

[69] Riley GA (1937) The significance of the Mississippi River drainage for biological conditions in the northern Gulf of Mexico. J Mar Res 1(1): 60–74.

[70] Dagg MJ, Breed GA (2003) Biological effects of Mississippi River nitrogen on the northern Gulf of Mexico–a review and synthesis. J Mar Syst 43(3): 133–152 10.1016/j.jmarsys.2003.09.002.

[71] Antoine JW (1972) Structure of the Gulf of Mexico. In: Rezak R, Henry VJ, editors. Texas A&M University Oceanographic Studies, Volume 3: Contributions on the geological and geophysical oceanography of the Gulf of Mexico. Houston: Gulf Publishing Company. 303 p.

[72] Pajuelo M, Bjorndal KA, Reich KJ, Vander Zanden HB, Hawkes LA, et al (2012b) Assignment of nesting loggerhead turtles to their foraging areas in the Northwest Atlantic using stable isotopes. Ecosphere 3(10): art89 10.1890/Es12-00220.1

[73] Troëng S, Evans DR, Harrison E, Lagueux CJ (2005) Migration of green turtles *Chelonia mydas* from Tortuguero,Costa Rica. Mar Biol 148(2): 435–447 10.1007/s00227-005-0076-4

[74] Rees AF, Saady SA, Broderick AC, Coyne MS, Papathanasopoulou N, et al (2010) Behavioural polymorphism in one of the world’s largest populations of loggerhead sea turtles *Caretta caretta* . Mar Ecol-Prog Ser 418: 201–212 10.3354/meps08767

[75] Schofield G, Hobson VJ, Lilley MKS, Katselidis K, Bishop CM, et al (2010) Inter-annual variability in the home range of breeding turtles: implications for current and future conservation management. Biol Cons 143(3): 722–730 10.1016/j.biocon.2009.12.011

[76] Weishampel JF, Bagley DA, Ehrhart LM (2004) Earlier nesting by loggerhead sea turtles following sea surface warming. Global Change Biol 10(8): 1424–1427 10.1111/j.1365-2486.2004.00817.x

[77] Lamont MM, Fujisaki I (2014) The effects of ocean temperature on nesting phenology and fecundity of the loggerhead turtle (*Caretta caretta*). J Herp 48(1): 98–102 10.1670/12-217

[78] Lagueux KM, Zani MA, Knowlton AR, Kraus SD (2011) Response by vessel operators to protection measures for right whales *Eubalaena glacialis* in the southeast US calving ground. Endanger Species Res 14: 69–77 10.3354/esr00335

[79] International Whaling Commission (IWC) (2008) Report of the sub-committee on estimation of bycatch and other human-induced mortality. J Cetacean Res Manage 10 (Supp.): 233–246.

[80] International Maritime Organization (IMO) (2008) New and amended existing traffic separation schemes (COLREG.2/Circ.60, Ref. T2-OSS/2.7.1). Available: www.imo.org/includes/blastDataOnly.asp/data_id%3D24700/60.pdf. Accessed 23 February 2010.

[81] U.S. Coast Guard (USCG) (2010) Port access route study: in the approaches to Los Angeles-Long Beach and in the Santa Barbara Channel. USCG, Washington, D.C. Federal Register 75 (68): 17562–17564 https://federalregister.gov/a/2010-7815.

[82] Hazel J, Lawler IR, Marsh H, Robson S (2007) Vessel speed increases collision risk for the green turtle *Chelonia mydas* . Endanger Spec Res 3: 105–113 10.3354/esr003105

[83] Zbinden JA, Aebischer A, Margaritoulis D, Arlettaz R (2008) Important areas at sea for adult loggerhead sea turtles in the Mediterranean Sea: satellite tracking corroborates findings from potentially biased sources. Mar Biol 153(5): 899–906 10.1007/s00227-007-0862-2

[84] Schofield G, Hobson VJ, Fossette S, Lilley MKS, Katselidis KA, et al (2010) Biodiversity Research: fidelity to foraging sites, consistency of migration routes and habitat modulation of home range by sea turtles. Divers Distrib 16(5): 840–853 10.1111/j.1472-4642.2010.00694.x

[85] Tucker AD, MacDonald BD, Seminoff JA (*In press)* Foraging site fidelity and stable isotope values of loggerhead turtles tracked in the Gulf Mexico and Northwest Caribbean. Mar Ecol-Prog Ser, doi:10.3354/meps10655.

[86] Schofield G, Scott R, Dimadi A, Fossette S, Katselidis KA, et al (2013) Evidence-based marine protected area planning for a highly mobile endangered marine vertebrate. Biol Conserv 161: 101–109 10.1016/j.biocon.2013.03.004

[87] Thomson JA, Heithaus MR, Burkholder DA, Vaudo JJ, Wirsing AJ, et al (2012) Site specialists, diet generalists? Isotopic variation, site fidelity, and foraging by loggerhead turtles in Shark Bay, Western Australia. Mar Ecol-Prog Ser 453: 213–226 10.3354/meps09637

[88] Hatase H, Matsuzawa Y, Sato K, Bando T, Goto K (2004) Remigration and growth of loggerhead turtles (*Caretta caretta*) nesting on Senri Beach in Minabe, Japan: life-history polymorphism in a sea turtle population. Mar Biol 144(4): 807–811 10.1007/s00227-003-1232-3

[89] Hawkes LA, Broderick AC, Coyne MS, Godfrey MH, Godley BJ (2007) Only some like it hot – quantifying the environmental niche of the loggerhead sea turtle. Divers Distrib 13(4): 447–457 10.1111/j.1472-4642.2007.00354.x

[90] Girard C, Tucker AD, Calmettes B (2009) Post-nesting migrations of loggerhead sea turtles in the Gulf of Mexico: dispersal in highly dynamic conditions. Mar Biol 156(9): 1827–1839 10.1007/s00227-009-1216-z

[91] Godley BJ, Blumenthal JM, Broderick AC, Coyne MS, Godfrey MH, et al (2008) Satellite tracking of sea turtles: Where have we been and where do we go next? Endang Species Res 4: 3–22 10.3354/esr00060

[92] USFWS and NMFS (2013) Endangered and Threatened Wildlife and Plants; Designation of Critical Habitat for the Northwest Atlantic Ocean Distinct Population Segment of the Loggerhead Sea Turtle (*Caretta caretta*) Federal Register 78. (57): 18000–18081.

[93] Bogdanova MI, Wanless S, Harris MP, Lindström J, Butler A, et al (2014) Among-year and within-population variation in foraging distribution of European shags *Phalacrocorax aristotelis* over two decades: implications for marine spatial planning. Biol Cons 170: 292–299.

[94] Schofield G, Dimadi A, Fossette S, Katselidis KA, Koutsoubas D, et al (2013) Satellite tracking large numbers of individuals to infer population level dispersal and core areas for the protection of an endangered species. Divers Distrib 19(7): 834–844.

[95] Phillips KF, Mansfield KL, Die DJ, Addison DS (2014) Survival and remigration probabilities for loggerhead turtles (*Caretta caretta*) nesting in the eastern Gulf of Mexico. Mar Biol. DOI:10.1007/s00227-013-2386-2.

[96] Meylan AB, Bjorndal KA, Turner BJ (1983) Sea Turtles Nesting at Melbourne Beach, Florida, II. Post-nesting Movements of *Caretta caretta* . Biol Cons 26: 79–90 10.1016/0006-3207(83)90050-2

[97] Dodd CK, Byles R (2003) Post-nesting movements and behavior of loggerhead sea turtles (*Caretta caretta*) departing from East-Central Florida nesting beaches. Chelonian Conserv Biol 4: 530–536.

[98] Hart KM, Sartain AR, Fujisaki I, Pratt HL Jr, Morley D, et al (2012) Home range, habitat use and migrations of hawksbill turtles tracked from Dry Tortugas National Park, Florida, USA. Mar Ecol-Prog Ser 457: 193–207 10.3354/meps09744

[99] Peterson CH, Anderson SS, Cherr GN, Ambrose RF, Anghera S, et al (2013) A Tale of Two Spills: Novel Science and Policy Implications of an Emerging New Oil Spill Model. BioScience 62(5): 461–469.

[100] Mitsch WJ, Day JW Jr, Gilliam JW, Groffman PM, Hey DL, et al (2001) Reducing Nitrogen Loading to the Gulf of Mexico from the Mississippi River Basin: Strategies to Counter a Persistent Ecological Problem: Ecotechnology–the use of natural ecosystems to solve environmental problems–should be a part of efforts to shrink the zone of hypoxia in the Gulf of Mexico. BioScience (2001) 51(5): 373–388 10.1641/0006-3568(2001)0510373:RNLTTG2.0.CO2

[101] Rabalais NN, Turner RE (2001) Hypoxia in the northern Gulf of Mexico: Description, causes and change. In: Coastal and Estuarine Studies Rabelais NN, Turner RE, editors. Coastal Hypoxia: Consequences for Living Resources and Ecosystems. Washington (DC): American Geophysical Union. 58: 1–36.

[102] Bograd SJ, Block BA, Costa DP, Godley BJ (2010) Biologging technologies: new tools for conservation. Endanger Species Res 10: 1–7 10.3354/esr00269

[103] Stewart KR, James MC, Roden S, Dutton PH (2013) Assignment tests, telemetry and tag-recapture data converge to identify natal origins of leatherback turtles foraging in Atlantic Canadian waters. J Anim Ecol 82: 791–803 10.1111/1365-2656.12056 23464527

[104] Parauka F, Duncan M, Lang P (2011) Winter coastal movement of Gulf of Mexico sturgeon throughout northwest Florida and southeast Alabama. J Appl Ichthyol 27(2): 343–350 10.1111/j.1439-0426.2011.01671.x

[105] Fertl D, Schiro AJ, Regan GT, Beck CA, Adimey N, et al (2005) Manatee occurrence in the Northern Gulf of Mexico, West of Florida. Gulf Caribb Res 17: 69–94.

[106] Leatherwood S (1975) Some observations of feeding behavior of bottle-nosed dolphins *(Tursiops truncatus)* in the Northern Gulf of Mexico and *(Tursiops* cf T. *gilli)* off Southern California, Baja California, and Nayarit, Mexico. Mar Fish Rev 37(9): 1157.

[107] Shaver DJ, Schroeder BA, Byles RA, Burchfield PM, Peña J, et al (2005) Movements and home ranges of adult male Kemp’s ridley sea turtles (*Lepidochelys kempii*) in the Gulf of Mexico investigated by satellite telemetry. Chelon Conserv Biol 4(4): 817–827.

[108] Hays GC, Fossette S, Katselidis KA, Schofield G, Gravenor MB (2010) Breeding periodicity for male sea turtles, operational sex ratios, and implications in the face of climate change. Conserv Biol 24(6): 1636–1643 10.1111/j.1523-1739.2010.01531.x 20497201

[109] Sonnier F, Teerling J, Hoese HD (1976) Observations on the offshore reef and platform fish fauna of Louisiana. Copeia: 105–111.

[110] Sammarco PW, Atchison AD, Boland GS (2004) Expansion of coral communities within the Northern Gulf of Mexico via offshore oil and gas platforms. Mar Ecol-Prog Ser 280: 129–143.

[111] Renaud ML, Carpenter JA (1994) Movements and submergence patterns of loggerhead turtles (*Caretta caretta*) in the Gulf of Mexico determined through satellite telemetry. Bull Mar Sci 55: 1–15.

[112] Work PA, Sapp AL, Scott DW, Dodd MG (2010) Influence of small vessel operation and propulsion system on loggerhead sea turtle injuries. J Exp Mar Biol Ecol 393(1): 168–175 10.1016/j.jembe.2010.07.019

[113] Halpern BS, Walbridge S, Selkoe KA, Kappel CV, Micheli F, et al (2008) A global map of human impact on marine ecosystems. Science 319: 948–952 10.1126/science.1149345 18276889

[114] Mawell SM, Hazen EL, Bograd SJ, Halpern BS, Breed GA, et al (2013) Cumulative human impacts on marine predators. Nat Commun 4: 2688 10.1038/ncomms3688 24162104

[115] Crouse DT, Crowder LB, Caswell H (1987) A stage-based population model for loggerhead sea turtles and implications for conservation. Ecology 68: 1412–1423.

[116] Heppell SS (1998) Application of life-history theory and population model analysis to turtle conservation. Copeia 1998: 367–375.

