# Supplementary figures and images for: Migration, Foraging, and Residency Patterns for Northern Gulf Loggerheads: Implications of Local Threats and International Movements
Source: PLoS One. 2014 Jul 30;9(7):e103453. doi: 10.1371/journal.pone.0103453 (PMC4116210; doi:10.1371/journal.pone.0103453)

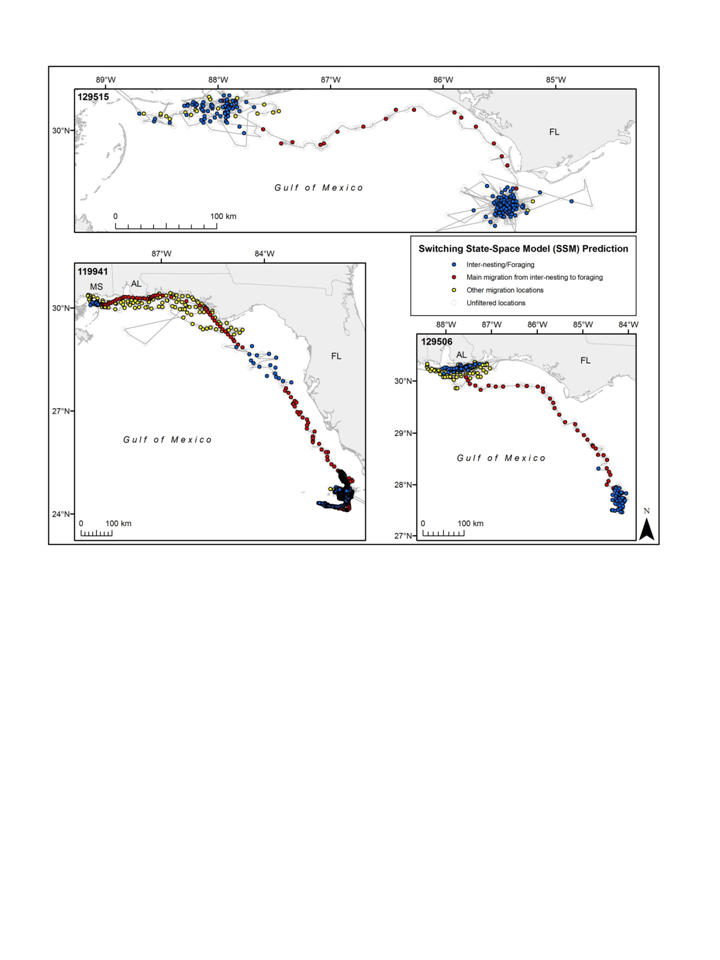

Supplement: Figure S1 — Predicted and actual tracks for Turtles 119941, 129506 and 129515 to show examples of exceptions for migration days (migration periods not directly before foraging periods, see Methods S1). Turtle 119941 had a small stop-over at-sea (blue points at-sea off NW Florida coast) in between migration (red points), and both the migration before and after were used. Turtle 129506 had two foraging periods as defined by our date cut-off (all blue points). The first was in the inter-nesting area near land and this period did not pass site fidelity. The visual inspection confirmed a ‘main’ migration after this first foraging period (red points). Turtle 129515 had a ‘main’ migration (red points) before an early small foraging period that was not the main foraging period used for analysis (blue points near St. Joseph Peninsula amidst other blue points used as foraging area. Yellow points in this area represent the small migration directly preceding the main foraging time). (TIF) [file pone.0103453.s001.tif]

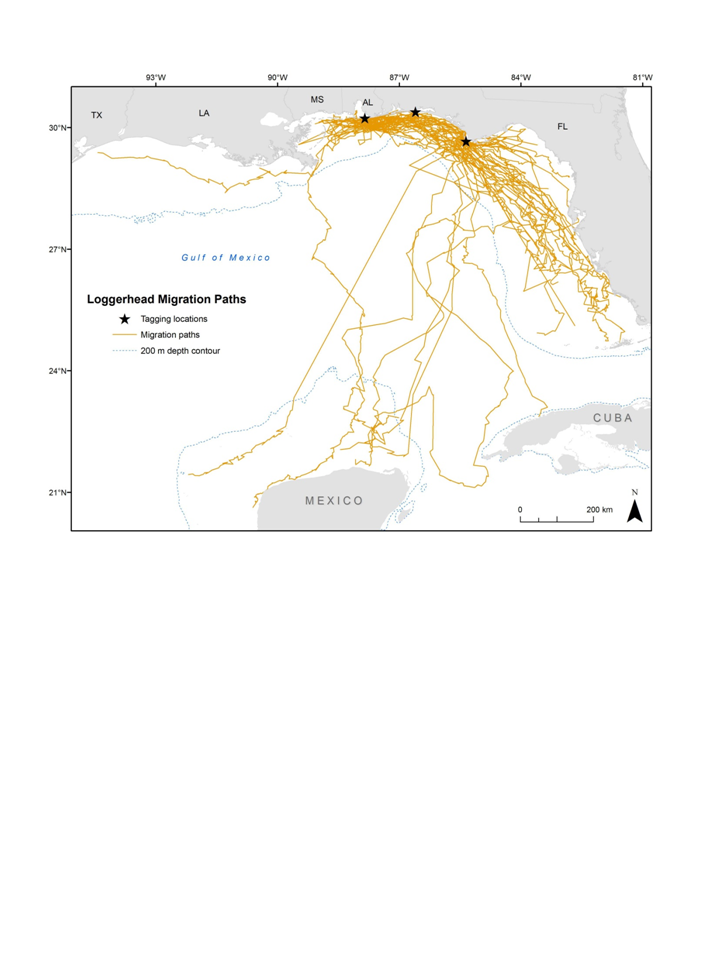

Supplement: Figure S2 — Migration tracks for 46 adult female loggerheads ( Caretta caretta ) after nesting in the Northern Gulf of Mexico 2010–2013. Tagging locations (black stars) from left to right are Gulf Shores, AL, Eglin Air Force Base, FL and St. Joseph Peninsula, FL. Lines created by connecting main migration locations (see Methods S1) filtered by swim speed with erroneous locations (land or very distant) removed. (TIF) [file pone.0103453.s002.tif]

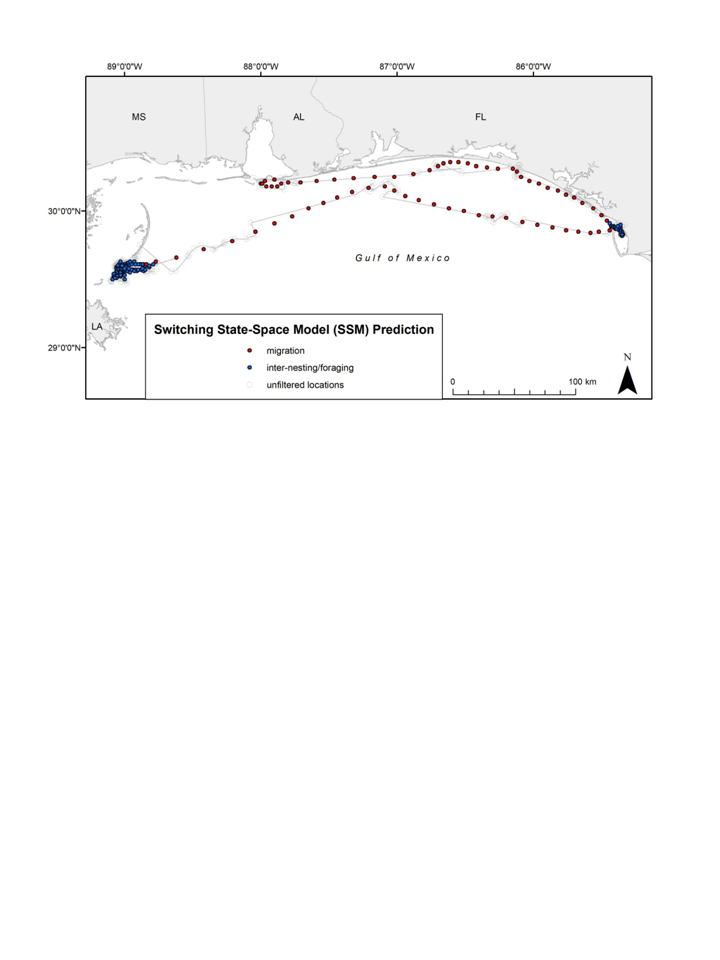

Supplement: Figure S3 — Example switching state-space model (SSM) prediction (red and blue points) over raw unfiltered locations (open grey circles) for Turtle119946 tagged in Gulf Shores, Alabama in 2012. (TIF) [file pone.0103453.s003.tif]

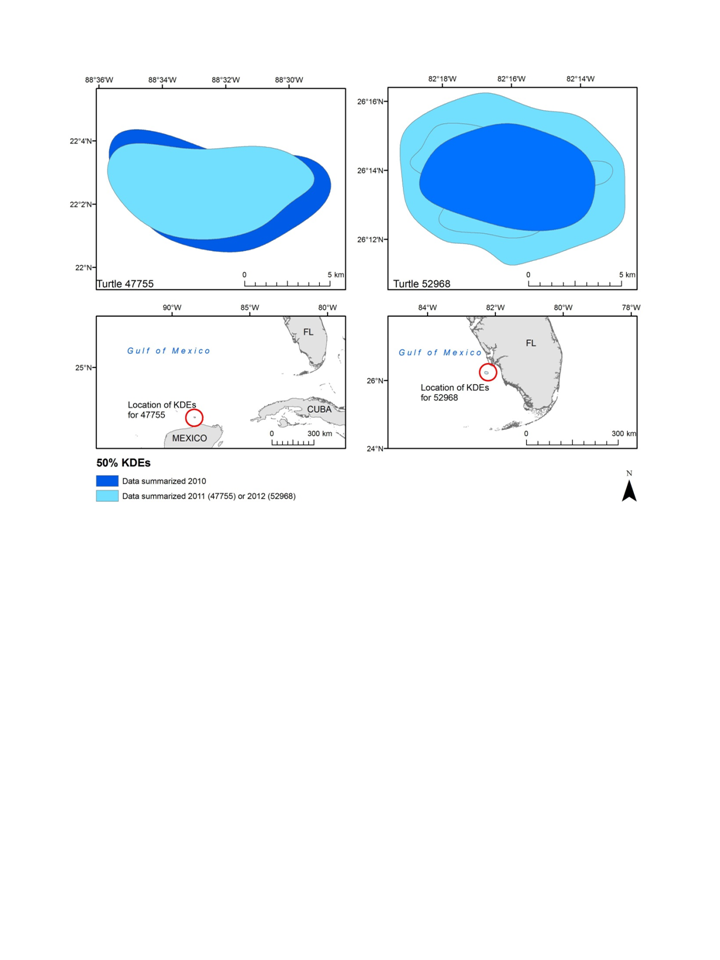

Supplement: Figure S4 — Foraging site kernel density estimate (KDE; 50%) for 2 adult female loggerheads ( Caretta caretta ) tagged in 2010 and previously summarized in Hart et al. (2012), shown in dark blue, and with an additional 489 days (turtle 52968) and 62 days (turtle 47755) of tracking data added during ‘foraging’ mode (light blue). (TIF) [file pone.0103453.s004.tif]

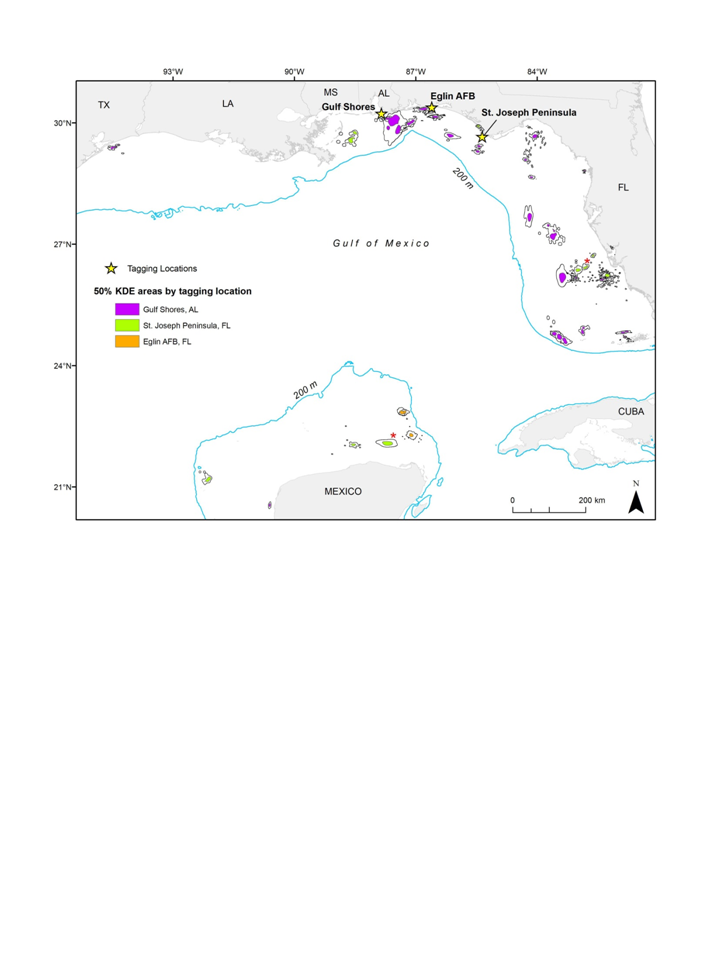

Supplement: Figure S5 — Foraging site kernel density estimates (KDE; 95% and 50%) for adult female loggerheads ( Caretta caretta ) that nested in the Northern Gulf at study sites in Alabama and Florida between 2010–2013. Red asterisks denote KDEs for 2 turtles from previous study (Hart et al. 2012). (TIF) [file pone.0103453.s005.tif]

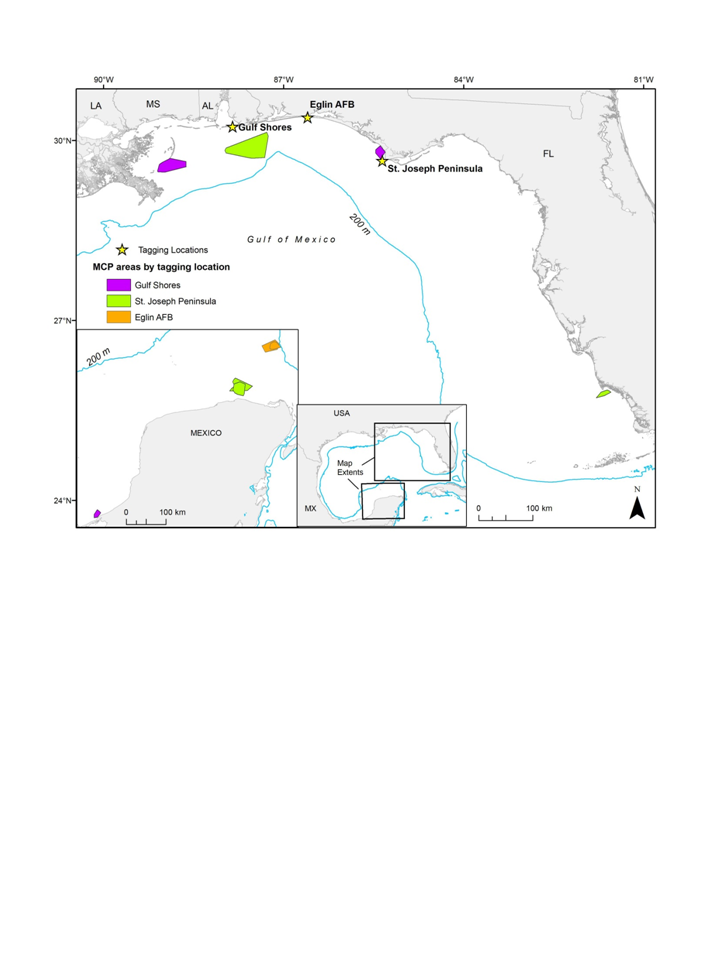

Supplement: Figure S6 — Ten minimum convex polygon (MCP) areas for 7 adult female loggerheads ( Caretta caretta ) satellite-tagged in the Northern Gulf (Turtle 129496 had 3 MCPs and Turtle 120439 had 2). MCPs are colored by tagging location. (TIF) [file pone.0103453.s006.tif]

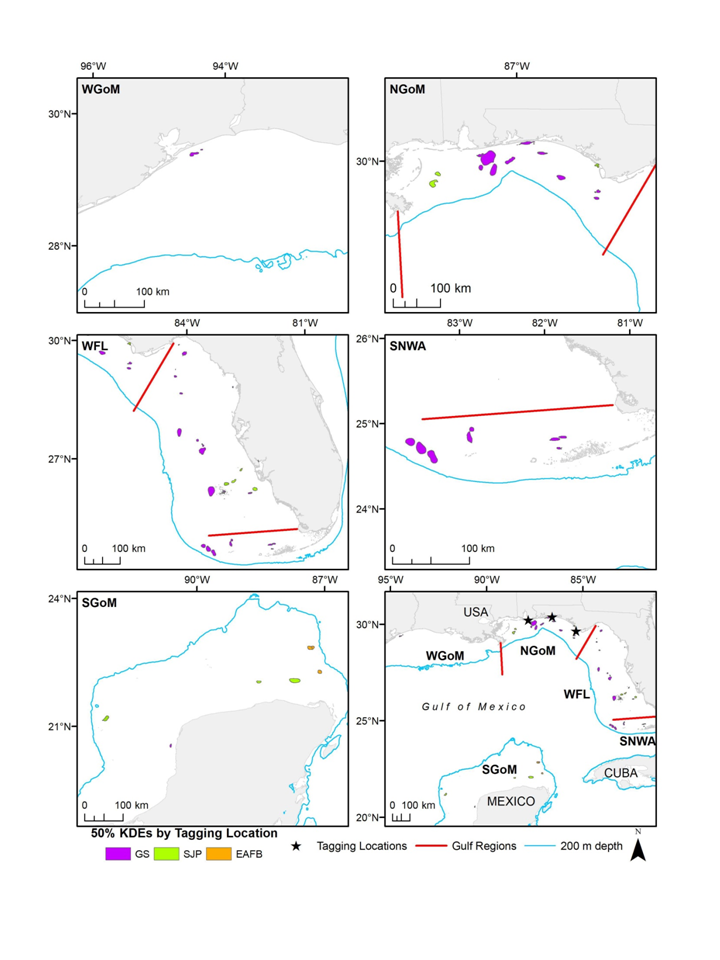

Supplement: Figure S7 — Kernel Density Estimation (KDE) for 38 adult female loggerheads (40 KDEs; Caretta caretta ) satellite-tagged in the Northern Gulf of Mexico (NGoM); contours are colored by original tagging location (GS = Gulf Shores, SJP = St. Joseph Peninsula, EAFB = Eglin Air Force Base). One turtle was tracked in both 2011 and 2012, and turtle 120439 had two foraging KDEs. Two turtles from Hart et al. (2012) are included. Gulf regions are denoted by red lines and are as follows: WGoM, Western Gulf of Mexico; NGoM; WFL, Western Florida; SNWA, Subtropical Northwest Atlantic; and SGoM, Southern Gulf of Mexico. (TIF) [file pone.0103453.s007.tif]
