# Supplementary material for: Migration, Foraging, and Residency Patterns for Northern Gulf Loggerheads: Implications of Local Threats and International Movements
Source: PLoS One. 2014 Jul 30;9(7):e103453. doi: 10.1371/journal.pone.0103453 (PMC4116210; doi:10.1371/journal.pone.0103453)
Supplement: Table S1 — Turtle info. (DOCX) [file pone.0103453.s008.docx]

| **Supporting Table 1.** Tags used by site and year. Grey areas indicate no tagging attempts at that site and year. A "-" indicates 0. | | | | | | |
| --- | --- | --- | --- | --- | --- | --- |
|  |  |  |  |  |  |  |
| **Location** | **Tag** | **2010** | **2011** | **2012** | **2013** | Total |
| **GS** | SPOT 5 | - | 8 | 10 | 5 |  |
|  | SPOT 5-MK10-AF | - | 5 | - | - |  |
|  | SPLASH | - | - | - | 9 | 37 |
| **SJP** | SPOT 5 | 3 | - | 1 | 5 |  |
|  | SPOT 5-MK10-AF | - | - | 5 | - |  |
|  | SPLASH | - | - | - | 1 |  |
|  | Kiwisat 101 PTT | 1 | - | 4 | - | 20 |
| **EAFB** | SPOT 5 | - | - | 2 | - | 2 |
|  | *Grand Total* | *4* | *13* | *22* | *20* | *59* |
|  |  |  |  |  |  |  |
| Kiwisat 101 PTT from Havelock North, New Zealand. | | | |  |  |  |
| All other tags from Wildlife Computers, Redmond, WA, USA | | | | |  |  |
