# Supplementary material for: Migration, Foraging, and Residency Patterns for Northern Gulf Loggerheads: Implications of Local Threats and International Movements
Source: PLoS One. 2014 Jul 30;9(7):e103453. doi: 10.1371/journal.pone.0103453 (PMC4116210; doi:10.1371/journal.pone.0103453)
Supplement: Table S2 — Tags used by site and year. (DOCX) [file pone.0103453.s009.docx]

| **Supporting Table 2.** Satellite-tracking details for adult foraging loggerheads in the Northern Gulf of Mexico. Last tracking date is either last location received or 10/15/2013, the time of data analysis, whichever is earlier. Tagging location given in italics. | | | |
| --- | --- | --- | --- |
|  |  |  |  |
| **Tag Number** | **Size (CCL-tip, cm)** | **Tagging Date** | **Last tracking date (days tracked)** |
| ***Gulf Shores, AL*** |  |  |  |
| 108170 | 95.2 | 6/6/2011 | 3/1/2012 (270) |
| 106360 | 92.3 | 6/7/2011 | 12/24/2011 (201) |
| 108172 | 93.9 | 6/8/2011 | 2/25/2012 (263) |
| 106345* | 90.1 | 6/9/2011 | 11/11/2011 (156) |
| 108171 | 90.4 | 6/9/2011 | 9/20/2012 (470) |
| 108173 | 95.9 | 6/11/2011 | 4/3/2012 (298) |
| 106337 | 93.6 | 6/11/2011 | 8/31/2011 (82) |
| 108174 | 88.0 | 6/13/2011 | 11/7/2011 (148) |
| 106358 | 92.5 | 6/14/2011 | 9/13/2011 (92) |
| 106361 | 92.0 | 6/15/2011 | 6/18/2012 (370) |
| 108961 | 91.5 | 7/23/2011 | 8/18/2011 (27) |
| 108964 | 94.6 | 7/30/2011 | 9/14/2011 (47) |
| 108965 | 87.0 | 7/31/2011 | 7/22/2012 (358) |
| 119940 | 94.2 | 6/2/2012 | 8/8/2012 (68) |
| 119941 | 92.0 | 6/3/2012 | 9/4/2013 (459) |
| 119943 | 97.5 | 6/4/2012 | 11/21/2012 (171) |
| 119938 | 97.5 | 6/4/2012 | 2/22/2013 (264) |
| 119924 | 95.4 | 6/6/2012 | 9/10/2012 (97) |
| 119944* | 90.8 | 6/7/2012 | 7/15/2013 (404) |
| 119946 | 95.0 | 6/9/2012 | 9/4/2012 (88) |
| 119945 | 98.9 | 6/9/2012 | 8/8/2012 (61) |
| 119923 | 95.6 | 6/13/2012 | 1/7/2013 (209) |
| 119947 | 98.5 | 6/13/2012 | 8/12/2012 (61) |
| 129502 | 86.1 | 6/4/2013 | 10/15/2013 (134) |
| 129501 | 96.8 | 6/8/2013 | 8/31/2013 (85) |
| 129503 | 94.5 | 6/8/2013 | 10/15/2013 (130) |
| 129504 | 92.6 | 6/10/2013 | 10/15/2013 (128) |
| 129505 | 95.6 | 6/10/2013 | 10/15/2013 (128) |
| 129506 | 96.1 | 6/11/2013 | 10/15/2013 (127) |
| 129507 | 99.0 | 6/11/2013 | 10/15/2013 (127) |
| 129508 | 97.3 | 6/11/2013 | 10/15/2013 (127) |
| 129509 | 90.4 | 6/12/2013 | 10/15/2013 (126) |
| 129510 | 97.0 | 6/14/2013 | 10/15/2013 (124) |
| 129512 | 104.3 | 6/16/2013 | 10/15/2013 (122) |
| 129513 | 93.4 | 6/17/2013 | 8/13/2013 (58) |
| 129511 | 98.0 | 6/19/2013 | 9/23/2013 (97) |
| 129515 | 100.4 | 6/23/2013 | 9/22/2013 (92) |
| ***St. Joseph Peninsula, FL*** | |  |  |
| 57656 | 99.5 | 7/26/2010 | 9/15/10 (52) |
| 89971 | 103.2 | 7/27/2010 | 9/16/10 (52) |
| 47755 | 97.2 | 8/3/2010 | 1/5/11 (156) |
| 52968 | 90.0 | 8/4/2010 | 3/7/12 (582) |
| 119942 | 82.4 | 6/10/2012 | 9/30/2012 (113) |
| 119948 | 92.5 | 6/11/2012 | 10/13/2012 (125) |
| 119952 | 90.1 | 7/23/2012 | 10/4/2012 (74) |
| 53017 | 88 | 6/3/2012 | 6/29/2012 (27) |
| 53016 | 102 | 6/5/2012 | 8/10/2012 (67) |
| 53164 | 102 | 6/8/2012 | 7/21/2012 (44) |
| 119949 | 100 | 6/11/2012 | 7/21/2012 (41) |
| 119950 | 102 | 6/11/2012 | 8/6/2012 (57) |
| 119952 | 101.1 | 6/13/2012 | 7/6/2012 (24) |
| 119951 | 103.3 | 6/22/2012 | 8/2/2012 (42) |
| 129496 | 90.1 | 6/11/2013 | 10/15/2013 (127) |
| 129497 | 96.5 | 6/12/2013 | 10/13/2013 (124) |
| 129498 | 93.1 | 6/13/2013 | 10/15/2013 (125) |
| 129499 | 101.6 | 6/13/2013 | 10/15/2013 (125) |
| 129500 | 90.6 | 6/14/2013 | 10/15/2013 (124) |
| 129514 | 87.3 | 7/10/2013 | 10/15/2013 (98) |
| ***Eglin AFB, FL*** |  |  |  |
| 120439 | 102.5 | 7/11/2012 | 5/24/2013 (318) |
| 120438 | 97.0 | 7/10/2012 | 10/15/2013 (463) |
| *mean-all turtles* | *95.0* |  | *156.4* |
| *SD-all turtles* | *4.9* |  | *126.9* |
| *mean-only AL turtles* | *94.4* |  |  |
| *SD-only AL turtles* | *3.8* |  |  |
| *mean-only FL turtles* | *96.0* |  |  |
| *SD-only FL turtles* | *6.3* |  |  |
