# Supplementary material for: Migration, Foraging, and Residency Patterns for Northern Gulf Loggerheads: Implications of Local Threats and International Movements
Source: PLoS One. 2014 Jul 30;9(7):e103453. doi: 10.1371/journal.pone.0103453 (PMC4116210; doi:10.1371/journal.pone.0103453)
Supplement: Table S3 — SSM prediction and model parameters for turtles 119946, 129515, 119941, and 129506. (DOC) [file pone.0103453.s010.doc]

**Table S3.** SSM prediction and model parameters for turtles 119946, 129515, 119941, and 129506

1. Turtle 119946

| **Node** | **Mean** | **SD** | **2.5% CI** | **97.5% CI** |
| --- | --- | --- | --- | --- |
| Process uncertainty from mode 1 to 1 (σ1,1) | 0.0049 | 0.0003 | 0.0043 | 0.0056 |
| Process uncertainty from mode 1 to 2 (σ1,2) | -0.0001 | 0.0002 | -0.0005 | 0.0003 |
| Process uncertainty from mode 2 to 1 (σ1,2) | -0.0001 | 0.0002 | -0.0005 | 0.0003 |
| Process uncertainty from model 2 to 2 (σ2,2) | 0.0031 | 0.0002 | 0.0027 | 0.0035 |
| Probability of being mode 1 (α1) | 0.9948 | 0.0025 | 0.9889 | 0.9985 |
| Probability of being mode 2 (α2) | 0.0443 | 0.0180 | 0.0171 | 0.0863 |
| Moving speed persistence in mode 1 (γ1) | 0.5852 | 0.0379 | 0.5106 | 0.6582 |
| Moving speed persistence in mode 2 (γ2) | 0.7704 | 0.0525 | 0.6679 | 0.8752 |

2. Turtle 129515

| **Node** | **Mean** | **SD** | **2.5% CI** | **97.5% CI** |
| --- | --- | --- | --- | --- |
| Process uncertainty from mode 1 to 1 (σ1,1) | 0.0083 | 0.0007 | 0.0072 | 0.0097 |
| Process uncertainty from mode 1 to 2 (σ1,2) | -0.0001 | 0.0003 | -0.0007 | 0.0004 |
| Process uncertainty from mode 2 to 1 (σ1,2) | -0.0001 | 0.0003 | -0.0007 | 0.0004 |
| Process uncertainty from model 2 to 2 (σ2,2) | 0.0043 | 0.0003 | 0.0038 | 0.0048 |
| Probability of being mode 1 (α1) | 0.9869 | 0.0064 | 0.9729 | 0.9966 |
| Probability of being mode 2 (α2) | 0.0500 | 0.0206 | 0.0163 | 0.0983 |
| Moving speed persistence in mode 1 (γ1) | 0.5199 | 0.0441 | 0.4295 | 0.6050 |
| Moving speed persistence in mode 2 (γ2) | 0.7245 | 0.0489 | 0.6227 | 0.8212 |

3. Turtle 119941

| **Node** | **Mean** | **SD** | **2.5% CI** | **97.5% CI** |
| --- | --- | --- | --- | --- |
| Process uncertainty from mode 1 to 1 (σ1,1) | 0.0134 | 0.0015 | 0.0106 | 0.0165 |
| Process uncertainty from mode 1 to 2 (σ1,2) | -0.0013 | 0.0006 | -0.0026 | -0.0002 |
| Process uncertainty from mode 2 to 1 (σ1,2) | -0.0013 | 0.0006 | -0.0026 | -0.0002 |
| Process uncertainty from model 2 to 2 (σ2,2) | 0.0064 | 0.0005 | 0.0054 | 0.0075 |
| Probability of being mode 1 (α1) | 0.9958 | 0.0024 | 0.9901 | 0.9993 |
| Probability of being mode 2 (α2) | 0.0157 | 0.0085 | 0.0041 | 0.0368 |
| Moving speed persistence in mode 1 (γ1) | 0.6931 | 0.0569 | 0.5746 | 0.7930 |
| Moving speed persistence in mode 2 (γ2) | 0.5480 | 0.0510 | 0.4458 | 0.6520 |

4. Turtle 129506

| **Node** | **Mean** | **SD** | **2.5% CI** | **97.5% CI** |
| --- | --- | --- | --- | --- |
| Process uncertainty from mode 1 to 1 (σ1,1) | 0.0058 | 0.0004 | 0.0051 | 0.0067 |
| Process uncertainty from mode 1 to 2 (σ1,2) | -0.0007 | 0.0002 | -0.0004 | 0.0003 |
| Process uncertainty from mode 2 to 1 (σ1,2) | -0.0007 | 0.0002 | -0.0004 | 0.0003 |
| Process uncertainty from model 2 to 2 (σ2,2) | 0.0032 | 0.0002 | 0.0029 | 0.0036 |
| Probability of being mode 1 (α1) | 0.9909 | 0.0050 | 0.9781 | 0.9978 |
| Probability of being mode 2 (α2) | 0.0204 | 0.0120 | 0.0060 | 0.0536 |
| Moving speed persistence in mode 1 (γ1) | 0.4172 | 0.0543 | 0.3117 | 0.5221 |
| Moving speed persistence in mode 2 (γ2) | 0.6373 | 0.0443 | 0.5588 | 0.7344 |
