# Supplementary material for: Migration, Foraging, and Residency Patterns for Northern Gulf Loggerheads: Implications of Local Threats and International Movements
Source: PLoS One. 2014 Jul 30;9(7):e103453. doi: 10.1371/journal.pone.0103453 (PMC4116210; doi:10.1371/journal.pone.0103453)
Supplement: Methods S1 — Detailed methods for why we selected or ruled out turtles for specific analyses and any exceptions made. (DOCX) [file pone.0103453.s011.docx]

SUPPLEMENTAL METHODS

One turtle was tracked twice (106345 and 119944), and was treated as two turtles for all summaries.

**2010 turtles for comparison**

Four turtles tracked in 2010 were added from Hart et al (2012) for comparison. The original KDE values and locations are referred to in this paper for two of these turtles (57656 and 89971). For these two, we used the existing KDE centroid locations to newly calculate the depth at the centroid locations. We used previously calculated distances to shoreline.

Two of these turtles, 52968 and 47755, had additional tracking days past their original analysis. These additional points all occurred near the previously determined foraging area, however we ran these tracks through SSM with the new points to confirm foraging grounds for the additional points and to therefore use all original and additional points for our foraging grid. We also created new KDE contours for these two turtles and followed the same methods for KDE creation and centroids as for all other turtles.

**SSM**

All turtles (n = 59) were attempted in SSM either in current or previous studies. Thirteen turtles were dropped from the SSM analysis however, due to too-short tracking durations or large transmission gaps.

Eight turtles (119941, 120438, 119938, 119944, 120439, 119923, 52968, 47755) were re-run in SSM because they continued transmitting after an initial analysis in previous years. These new points/dates were used in a new KDE analysis.

**Foraging periods**

SSM results include two modes: migration and non-migration. In order to differentiate whether non-migration periods were inter-nesting or foraging, we used the date of August 15 as the cut-off between them. Therefore, from the ‘inter-nesting and/or foraging’ modes, we determined a period to be ‘inter-nesting’ (and thus not used for this analysis), if all points in that period occurred solely up to and including August 15 of the nesting year. All other subsequent non-migration periods were considered to be representative of foraging behavior.

We made an exception for one turtle that departed the study area earlier than other turtles, as her SSM data showed a clear transition into migration mode followed by a second clear transition to foraging mode prior to the August 15 cut-off. For this turtle (129513), we used foraging locations beginning July 22.

**MCP exception**

Of the 13 turtles for which SSM did not successfully predict a plausible path, turtle 108171 showed a possible foraging period upon visual inspection of the transmitted locations. This turtle did not have a successful SSM run due to long data gaps. However, the first point after the long gap was on September 4 and within a cluster of points off the coast of Mexico. All points thereafter remained in that area, during a time in which the turtle would likely be in foraging mode. These points were therefore considered to be a potential foraging period. The raw data was filtered as for other turtles, and the remaining points (from 4 September 2012 until 20 September 2012) were used in MCP analysis.

**Migrations**

The SSM identified multiple migration times for turtles, some within timeframes considered to be inter-nesting and foraging. To determine which migration periods were the ‘main’ migration between inter-nesting and foraging areas, and use these dates in our analyses, we visually inspected the paths.

We found that the migration time directly preceding the foraging area (determined through SSM and our cut-off date of 8/15) represented the ‘main’ migration for all but three turtles: 119941 had a small stop-over at-sea in between migration, and both the migration before and after were used; 129506 had two foraging periods as defined by our date cut-off. The first was in the inter-nesting area near land and this period did not pass site fidelity. The visual inspection confirmed a ‘main’ migration after this first foraging period; 129515 had a ‘main’ migration before an early small foraging period that was not the main foraging period used for analysis.

For the Hart et al. (2012) turtles, migrations dates for the two that we ran in a current SSM were the same as for all other turtles. Migration dates for the two that were not run in another SSM were taken from Hart et al. (2012).

One turtle (129501) with a successful SSM run did not have a foraging period, as transmission ceased shortly after this turtle migrated away from the inter-nesting area and reached the coastline of Cuba (presumed foraging area). We included these migration points as a “main” migration, even though this turtle had no foraging period.

**Last points**

We plotted the last transmitted, filtered location for turtles with foraging periods that did not pass site fidelity and for those dropped from SSM, except for turtle 108171 for which we made an MCP (see MCP exception above).
